# Long-term quality of life, rehospitalization risks, and economic viability of remote cardiac rehabilitation: an updated systematic review and meta-analysis

**DOI:** 10.3389/fdgth.2026.1935832

**Published:** 2026-10-14

**Authors:** Hui Pei, Hengrui Li, Miaomiao Hu, Boyan Liu, Dali Hu, Yuanmin Li, Qiang Xiao

**Affiliations:** 1 Department of Cardiology, The Second Affiliated Hospital of Shandong First Medical University, Taian, China; 2 Department of Rehabilitation Medicine, Qingdao Central Hospital, University of Health and Rehabilitation Sciences, Qingdao, China; 3 Department of Pediatrics, The Second Affiliated Hospital of Shandong First Medical University, Taian, China; 4 Taishan Institute for Hydrogen Biomedical Research, The Second Affiliated Hospital of Shandong First Medical University, Tai'an, China; 5 Department of Ultrasonography, The Second Affiliated Hospital of Shandong First Medical University, Taian, China; 6 Shandong Provincial Key Medical and Health Discipline of Emergency Medicine, The Second Affiliated Hospital of Shandong First Medical University, Taian, China

**Keywords:** cardiac rehospitalization, health-related quality of life, remote cardiac rehabilitation, systematic review and meta-analysis, trial sequential analysis

## Abstract

**Background:**

Although remote cardiac rehabilitation (RCR) is a promising alternative to traditional center-based care, evidence regarding its exact impact on health-related quality of life (HRQoL), cardiovascular safety, and economic viability remains fragmented, necessitating a comprehensive synthesis to guide clinical implementation.

**Objectives:**

This systematic review and meta-analysis aim to evaluate the HRQoL, cardiovascular safety, and economic viability of RCR versus standard center-based care in cardiovascular disease, with subgroup analyses based on follow-up duration and high-risk patient cohorts.

**Methods:**

Literature searches were conducted across multiple databases, including PubMed, Embase, and the Cochrane Library, supplemented by manual screening of relevant references to identify clinical studies on RCR up to July 2026. Two independent review teams performed data extraction and risk of bias assessment using the RevMan software. Meta-analyses were conducted employing random-effects models to calculate pooled effect sizes with corresponding 95% confidence intervals. Heterogeneity was assessed using the Cochrane Q test and *I*^2^ statistic, and potential publication bias was evaluated using funnel plots and Egger's test. To evaluate the robustness of the pooled results and explore potential heterogeneity, subgroup and fixed-effects sensitivity analyses were conducted, supplemented by trial sequential analysis (TSA).

**Results:**

A total of 45 randomized controlled trials were included in this meta-analysis. RCR significantly improved HRQoL across multiple scales (MacNew: MD = 0.17; MLHFQ: MD = −5.38; *P* < 0.05), while maintaining SF-36 and HADS outcomes comparable to standard care. Exploratory analyses indicated statistically significant improvements in mental health at 6 months (MD = 1.73, *P* = 0.03) and physical functioning at 12 months (MD = 1.48, *P* = 0.0002). Regarding cardiovascular safety, RCR was not associated with a statistically significant increase in the risk of MACEs, all-cause mortality, or adverse events (*P* > 0.05) compared to conventional care. RCR significantly reduced the risk of cardiac rehospitalization (RR = 0.70, *P* = 0.01), with exploratory stratified analyses indicating that this favorable association was evident in the hardware-integrated interventions subgroup (RR = 0.59, *P* = 0.02) and 6-month observation subgroup (RR = 0.38, *P* < 0.00001), alongside a non-significant numerical protective trend among high-risk patients (RR: 0.59 vs. 0.74, *P* = 0.15). The economic viability of RCR remains uncertain, critically depending on initial setup outlays, patient scaling, and subsequent healthcare utilization.

**Conclusions:**

RCR may serve as an effective and safe alternative to conventional care that improves physical and psychological well-being, was associated with a lower risk of cardiac rehospitalization, and may provide economic value in certain healthcare systems.

**Systematic Review Registration:**

https://www.crd.york.ac.uk/PROSPERO/view/CRD420261374977, PROSPERO CRD420261374977.

## Introduction

Cardiac rehabilitation (CR), a comprehensive intervention for secondary cardiovascular prevention, is widely recommended by major international guidelines as a cornerstone of care in patients with cardiovascular disease (1, 2). Extensive clinical evidence has established the critical role of cardiac rehabilitation in mitigating disease progression, reducing mortality, and improving long-term prognosis. Despite this strong evidence and guideline support, the real-world impact of traditional center-based CR remains severely limited, with actual participation rates estimated at merely 20% to 30%, compounded by poor adherence (3). This clinical underutilization is primarily driven by geographic barriers, resource limitations, and substantial financial and time costs for patients, underscoring an urgent need for alternative delivery models (4).

Remote cardiac rehabilitation (RCR) has emerged as a promising solution to address these barriers (5). By shifting rehabilitation into the home environment, RCR utilizes telemonitoring, mobile health applications, video consultations, and telephone support to deliver comprehensive interventions (6–8). This model improves accessibility, enhances patient participation, and reduces travel and time burdens, making it particularly suitable for patients with low- to moderate-risk cardiovascular disease and those living in resource-limited or geographically remote areas.

Previous small-sample clinical studies and meta-analyses (5, 9, 10) have shown that RCR achieves high patient adherence and effectively improves exercise capacity, supporting its feasibility as an important adjunct to center-based rehabilitation. Beyond physiological benefits, RCR can also enhance mental health and quality of life by reducing psychological burden. This psychological improvement has crucial clinical significance, as stress, anxiety, and depression are recognized independent prognostic risk factors for adverse cardiovascular events. Nonetheless, differences in HRQoL instruments (such as the SF-36 and MacNew scales) across studies complicate direct comparisons and limit the interpretability of existing findings.

Despite these promising functional outcomes, the safety of RCR remains a major concern, particularly for high-risk patients with severe cardiovascular disease. The transition to a home-based setting inherently reduces face-to-face medical supervision and deprives clinicians of routine physical examinations. The reliance on remote devices introduces potential measurement errors; such limitations in continuously tracking physiological changes may obscure subtle signs of clinical deterioration and impede real-time adjustments to treatment plans. Most critically, the absence of immediate on-site medical intervention severely compromises emergency responsiveness during acute exercise-induced events, potentially delaying life-saving interventions.

Several clinical studies support digitally-delivered cardiac rehabilitation, existing meta-analyses have primarily focused on functional efficacy and short-term patient adherence (5, 9, 10). The recent network meta-analysis confirmed the superiority of specific telehealth combinations in maximizing peak oxygen uptake (SUCRA=86.8%) and patient adherence (SUCRA=74.2%), but left long-term cardiovascular safety unaddressed. There remains a critical evidence gap concerning the long-term cardiovascular safety across diverse risk profiles, alongside the impact on HRQoL, particularly regarding psychosocial and mental health outcomes. This evidentiary deficit is especially pronounced among high-risk patients with severe cardiovascular disease, a population that has been marginalized in previous research and currently lacks defined optimal management strategies.

Therefore, this meta-analysis aims to address these limitations by synthesizing high-quality randomized controlled trial data to evaluate HRQoL, hard clinical safety endpoints, and economic viability. By incorporating rigorous subgroup analyses across cardiovascular risk profiles, intervention delivery modalities, and follow-up durations, this study seeks to evaluate the clinical associations and safety profiles of RCR, providing the evidence base required to optimize RCR implementation, guide personalized intervention strategies, and inform evidence-based health policy.

## Methods

### Protocol and registration

The systematic review and meta-analysis were conducted and reported in strict accordance with the Preferred Reporting Items for Systematic Reviews and Meta-Analyses (PRISMA) guidelines and the Cochrane Handbook for Systematic Reviews of Interventions. The protocol for this review was prospectively registered in the PROSPERO database (CRD420261374977).

### Literature search strategy

The literature search was conducted across multiple databases, including PubMed, Embase, and the Cochrane Library, covering the period from database inception to July 2026. Search terms were developed based on the key components of the research question, incorporating both relevant keywords and controlled vocabulary, and the full search strategy is provided in the Supplementary Material. Additionally, grey literature sources such as conference proceedings and clinical trial registries were searched to capture unpublished studies. Manual screening of reference lists from eligible articles and relevant systematic reviews was also performed to supplement the literature search and enhance comprehensiveness.

### Study selection

The study selection process was conducted independently by two reviewers to ensure methodological rigor and reproducibility. After removing duplicate records across all databases using EndNote X9 software, titles and abstracts were initially screened to exclude clearly ineligible studies. Subsequently, the full texts of all potentially relevant articles were retrieved and meticulously assessed against the established PICOS framework. Any disagreements between reviewers were resolved through consensus discussion, and when necessary, by consultation with a third reviewer. Finally, the number of records identified, excluded at each stage, and included in the review was summarized in a PRISMA flow diagram to enhance transparency and clarity.

### Eligibility and exclusion criteria

Eligibility criteria were established *a priori* based on the PICOS framework. This review was restricted to RCTs evaluating RCR in patients with established cardiovascular disease. The intervention of interest was any RCR program delivered outside hospital settings using telemonitoring, mobile applications, video consultations, or telephone-based follow-up, compared with usual care, center-based cardiac rehabilitation, or another form of standard management. Eligible studies were required to report at least one of the following prespecified outcomes: health-related quality of life (HRQoL), cardiovascular safety profiles (e.g., MACEs, mortality, adverse events), healthcare utilization (e.g., readmission rates), or health economic evaluations. Exclusion criteria included non-randomized study designs, animal studies, pediatric populations, and studies that did not report data on any of the prespecified outcomes.

### Outcomes

The primary outcome of this meta-analysis was the change in HRQoL, evaluated using a comprehensive four-scale framework: the 36-Item Short Form Survey (SF-36), the MacNew Heart Disease Health-related Quality of Life Questionnaire, the Minnesota Living with Heart Failure Questionnaire (MLHFQ), and the Hospital Anxiety and Depression Scale (HADS).

Secondary outcomes were categorized into clinical safety endpoints and health economic evaluations. Clinical and safety endpoints encompassed MACEs, all-cause mortality, all-cause readmissions, and cardiac hospitalization. Health economic outcomes assessed the financial viability and cost-effectiveness of the remote interventions, incorporating metrics such as total healthcare expenditures and the Incremental Cost-Effectiveness Ratio (ICER).

### Data extraction

Extracted data systematically encompassed four main domains: (1) study characteristics (first author, publication year, country, design, and registration number); (2) participant demographics (sample size, age, sex, diagnosis, and risk profiles); (3) RCR treatment details (intervention mode, duration, and specific comparators); and (4) outcome measures (predefined criteria for HRQoL and adverse events, as well as follow-up duration). To maintain data accuracy and reliability, any discrepancies between the two reviewers were resolved through consensus discussion or consultation with a third reviewer.

### Quality assessment and risk of bias

Quality assessment and risk of bias for the included studies were evaluated using the Cochrane Risk of Bias Tool (RoB 1), which encompasses several domains including the type of analysis, random sequence generation, allocation concealment, blinding of participants and personnel, and blinding of outcome assessment, each of which was judged as “low risk,” “unclear risk,” or “high risk”. These domains were assessed independently by two reviewers to minimize the risk of bias in the evaluation.

### Statistical synthesis and model selection

Meta-analysis was performed using RevMan (version 5.3) and Stata (version 17.0) following Cochrane Handbook guidelines. To conservatively account for the methodological heterogeneity across the included trials, a random-effects model was prospectively applied as the primary analytical approach for all pooled effect estimates, alongside a comparative evaluation against a fixed-effects model. To rigorously test the robustness of our findings, comprehensive sensitivity analyses were subsequently conducted across all included studies using a strict fixed-effects model. Dichotomous outcomes were summarized as risk ratios (RRs) calculated via the Mantel-Haenszel method, while continuous outcomes were expressed as mean differences (MDs) utilizing the inverse-variance method, both with corresponding 95% confidence intervals (CIs) constructed using the standard Wald-type method based on a normal distribution. Between-study variance (τ^2^) in the random-effects models was estimated using the DerSimonian and Laird (DL) method.To appropriately handle sparse data or trials with a zero event in a single arm, a standard continuity correction of 0.5 was added to each cell of the 2 × 2 contingency table to facilitate their inclusion in the pooled analysis. Studies with zero events in both treatment and control arms were excluded from the pooled relative effect estimation, in accordance with standard Cochrane methodological practices.

### Subgroup and stratification analyses

Subgroup analyses were performed to explore potential sources of heterogeneity, comprising both prespecified and exploratory *post-hoc* analyses based on the following clinical and methodological characteristics:

1. Cardiovascular Risk Profile: To ensure an objective and reproducible stratification, study cohorts were categorized using predefined quantitative and clinical thresholds. Cohorts were classified as “high-risk ” if they met at least one of the following explicit criteria: a mean or median baseline LVEF ≤ 40% (or >50% of the study population exhibiting an LVEF≤40%); > 30% of participants classified as NYHA functional class III–IV (or >50% as NYHA class II–IV); or those requiring cardiac implantable electronic devices [CIEDs]). Conversely, cohorts not meeting any of these high-risk thresholds were explicitly classified into the “low-risk” group.
2. Intervention Modality: To evaluate the influence of technological architecture and telemonitoring complexity, an exploratory *post-hoc* subgroup analysis categorized digital RCR programs by intervention modality: Basic telecommunication RCR, relying on conventional, low-tech communication tools such as structured telephone follow-up or SMS text messaging without remote sensor support; Software-only digital RCR, utilizing standalone mobile applications or web platforms for patient education, lifestyle self-logging, and communication without peripheral hardware integration; and Hardware-integrated digital RCR, incorporating wearable activity trackers, Bluetooth-enabled physiological monitoring devices, or tele-ECG IoT systems enabling objective and automated telemonitoring.
3. Follow-up Duration: To evaluate the sustainability of the intervention effects, the outcome assessments were stratified into three distinct temporal subgroups: short-term (< 6 months), medium-term (∼ 6 months), and long-term (> 6 months).

### Assessment of publication bias and trial sequential analysis

Potential publication bias was evaluated through visual inspection of funnel plots alongside Egger's linear regression test for outcomes with at least 10 included trials. To further assess the robustness of the cumulative evidence and minimize the risk of type I errors due to repeated significance testing, trial sequential analysis (TSA) was performed using TSA software version 0.9.5.10 Beta (Copenhagen Trial Unit). The required information size (RIS) and trial sequential monitoring boundaries were constructed based on empirical effect estimates, maintaining an overall two-sided *α* of 0.05 with a statistical power of 80%.

## Result

### Study characteristics

There were 622 records identified through database searches, including 256 from Embase, 251 from MEDLINE, and 115 from the Cochrane Library. After removing 164 duplicate records, 458 related records remained for title and abstract screening process. Following title and abstract screening, 386 records were excluded due to unrelated topics, non-RCT designs, animal studies, reviews, conference abstracts, and case reports, leaving 72 articles for full-text assessment. After detailed full-text review, 27 articles were further excluded due to inconsistent interventions or outcomes and lack of necessary research data. Finally, 45 randomized controlled studies were included in this systematic review and meta-analysis (9, 11–55). The study selection process is illustrated in the PRISMA flow diagram (Figure 1A), while the risk of bias assessments for each included trial was presented in Figure 1B. The study characteristics of included studies were summarized in Supplementary Table.

**Figure 1:**
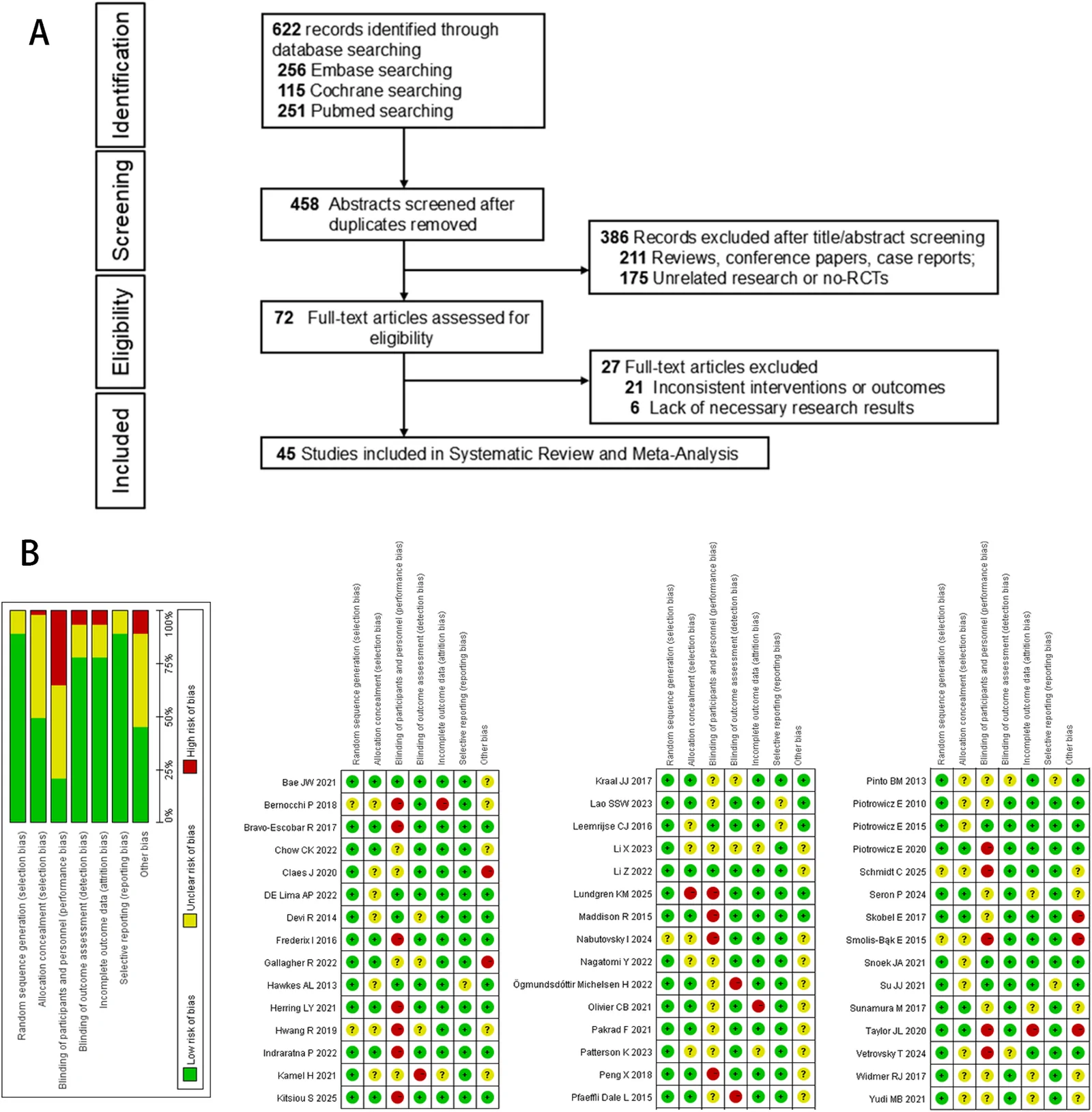
The PRISMA flowchart and the risk assessment of the included studies. **(A)** The PRISMA flowchart of this study; **(B)** Results of risk assessment of 45 randomized controlled studies.

### Health-related quality of life

To comprehensively assess the impact of RCR on health-related quality of life (HRQoL) in cardiovascular patients, this meta-analysis employed a four-scale framework, including the MacNew Questionnaire, the Minnesota Living with Heart Failure Questionnaire (MLHFQ), the 36-Item Short Form Survey (SF-36), and the Hospital Anxiety and Depression Scale (HADS). The meta-analysis of 12 RCTs (9, 14, 17, 34, 38–40, 42, 43, 45, 46, 56) demonstrated that RCR conferred significant improvements in HRQoL compared to CR, as evidenced by the MacNew (MD = 0.17, *P* < 0.00001) and MLHFQ (MD = −5.38, *P* = 0.03) scores in Figures 2A, B. While, the SF-36 scores remained stable between the interventions, with no statistically significant difference (MD = 1.39, 95% CI: −1.06 to 3.84, *P* = 0.27) in Figure 2C.

**Figure 2:**
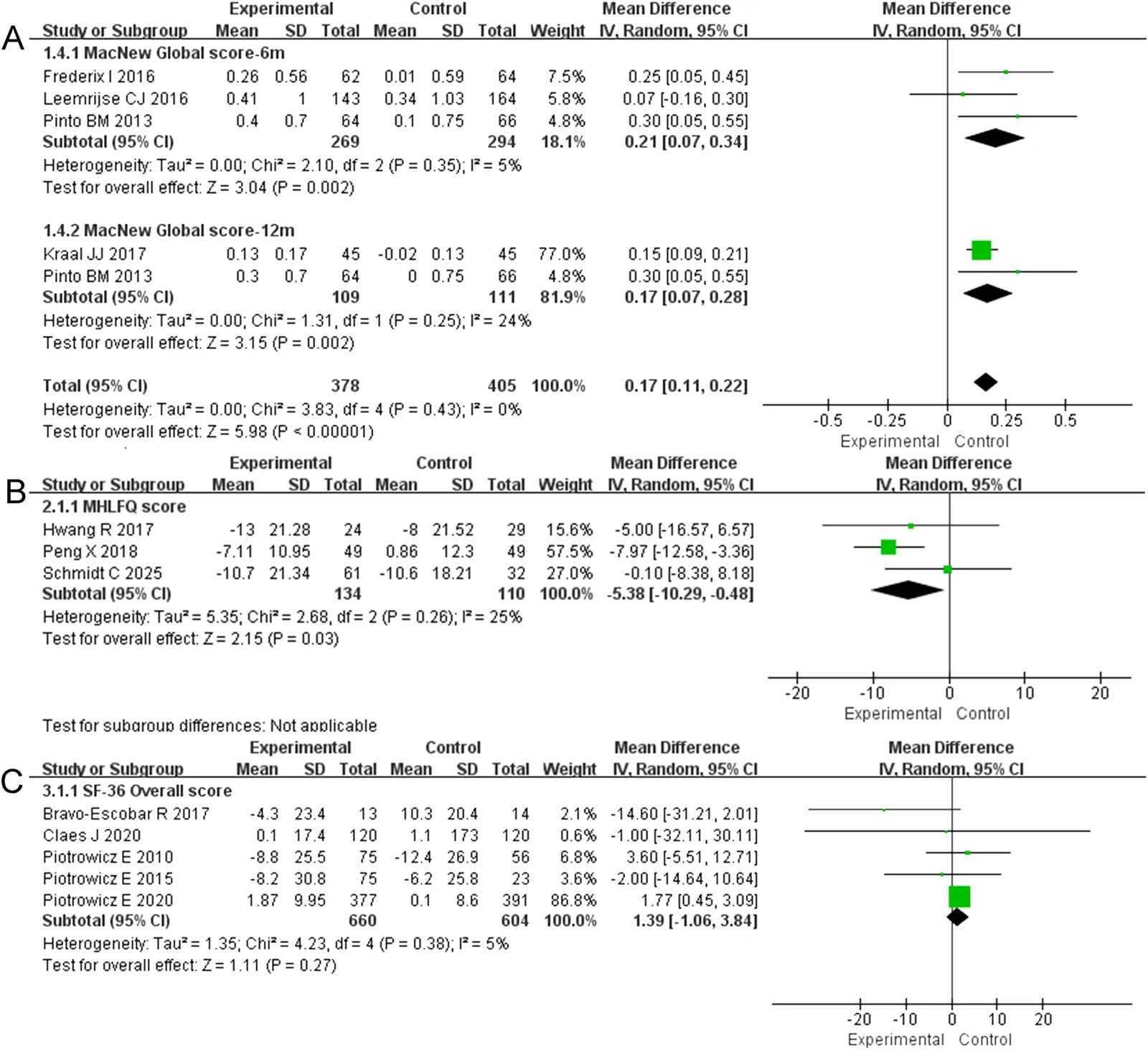
Forest plots of the effects of RCR on HRQoL as measured by three scales: **(A)** MacNew global score; **(B)** MLHFQ score; **(C)** SF-36 overall score.

Regarding psychological and emotional well-being, the RCR effects varied slightly across scales but demonstrated a generally consistent trend. The meta-analysis of the SF-36 scale indicated statistically significant improvements in mental health at 6 months (MD = 1.73, *P* = 0.03), but no significant differences at 12 months (MD = −0.10, *P* = 0.91), as shown in Figure 3A. Similarly, the MacNew emotional score suggested that RCR conferred early psychological benefits at 6 months (MD = 0.16, *P* = 0.08), which were sustained through the 12-month follow-up (MD = 0.35, *P* < 0.0001) in Figure 3B. The HADS revealed no statistically significant difference between the interventions (Supplementary Material).

**Figure 3:**
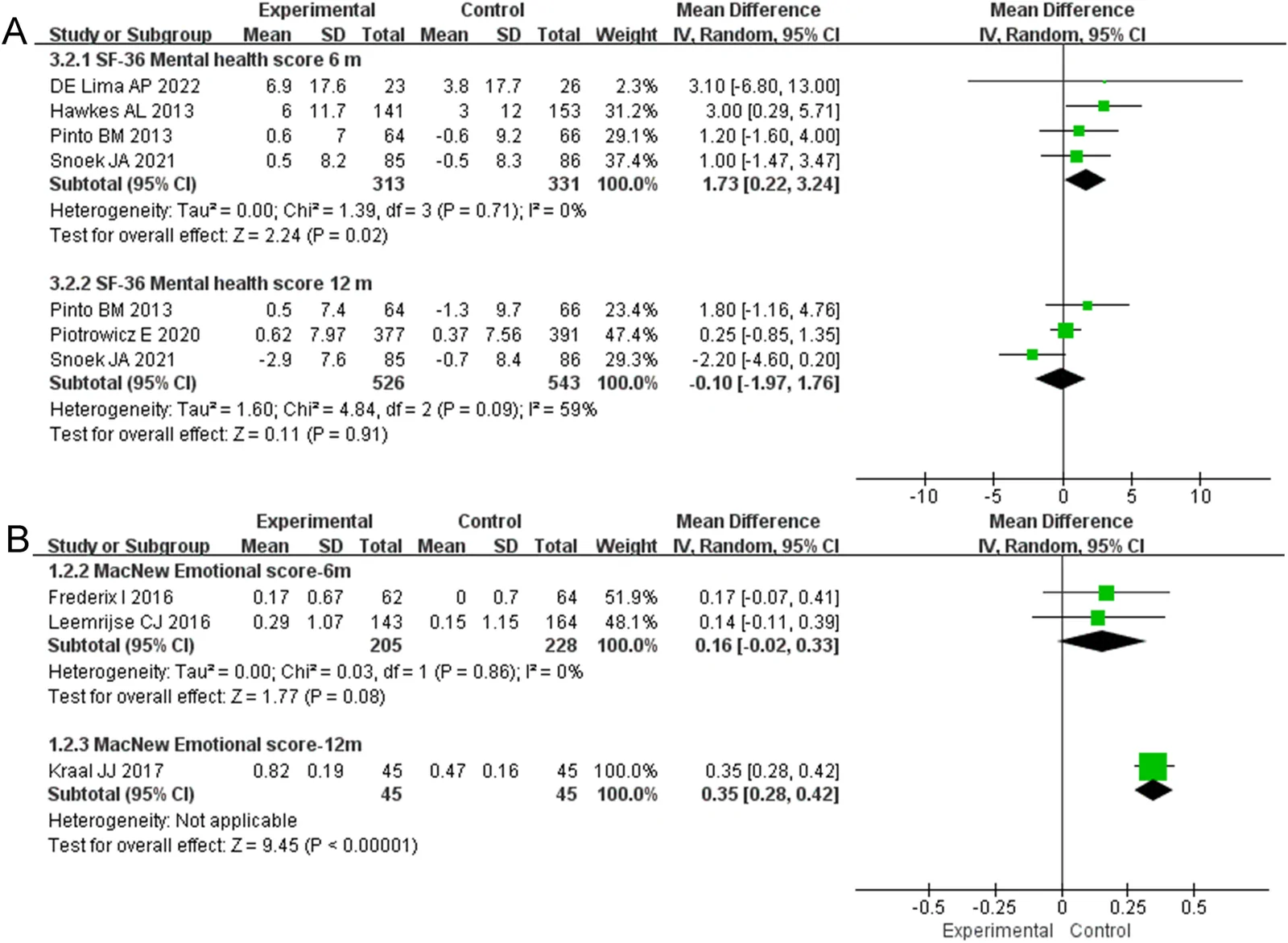
Forest plots of the effects of RCR on psychological and emotional well-being as measured by the SF-36 and MacNew scales. **(A)** SF-36 mental health score at 6 and 12 months; **(B)** MacNew emotional score at 6 and 12 months.

Regarding physical functional recovery, the advantages of RCR manifest as a cumulative, time-dependent process that yields profound long-term benefits beyond 12 months. Meta-analysis of the physical functioning domains on the SF-36 (MD = 0.43, *P* = 0.60) and MacNew (MD = 0.21, *P* = 0.01) scales revealed minimal to modest differences between RCR and CR at the 6-month follow-up (Supplementary Material). However, beyond 12 months of follow-up, three RCTs (9, 17, 26) confirmed that the therapeutic efficacy of RCR on physical function expanded substantially, demonstrating highly significant improvements over CR (SF-36: MD = 1.48, *P* = 0.0002; MacNew: MD = 0.35, *P* < 0.00001) in Figures 4A, B.

**Figure 4:**
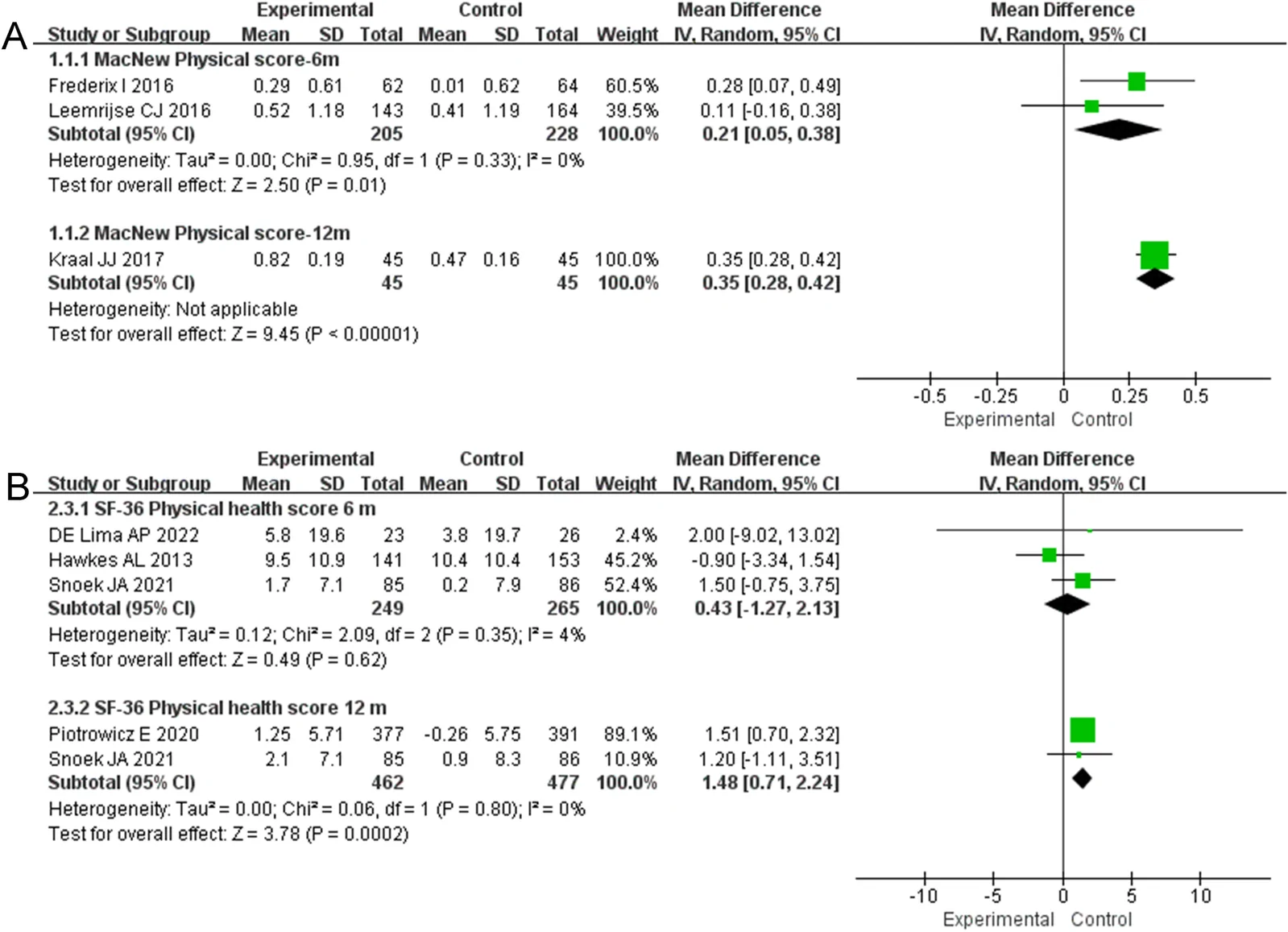
Forest plots of the effects of RCR on physical functional recovery as measured by the MacNew and SF-36 scales. **(A)** MacNew physical score at 6 and 12 months; **(B)** SF-36 physical health score at 6 and 12 months.

### Mortality, hospitalization, and safety outcomes

Regarding clinical safety, seventeen RCTs (9, 11, 13, 14, 16, 19, 20, 23, 26, 28, 30–34, 48, 52, 53) were included in the meta-analysis of MACEs, all-cause mortality, and all-cause adverse events. Meta-analysis showed that RCR did not increase the incidence of MACEs (RR = 0.98, 95% CI: [0.80, 1.17], *P* = 0.80), all-cause mortality (RR = 1.02, *P* = 0.91), or all-cause adverse events (RR = 1.12, *P* = 0.36) compared to conventional CR during the follow-up period (Supplementary Material). Although the meta-analysis of 21 studies (9, 13, 15, 17, 18, 20–22, 27, 29–33, 43, 47, 51–55) indicated only a marginal trend toward lower all-cause readmission risk with RCR (RR = 0.91, 95% CI: [0.82, 1.01], *P* = 0.08), it revealed a statistically significant reduction in cardiac rehospitalization (RR = 0.70, 95% CI: [0.52, 0.93], *P* = 0.01) in Figure 5A.

**Figure 5:**
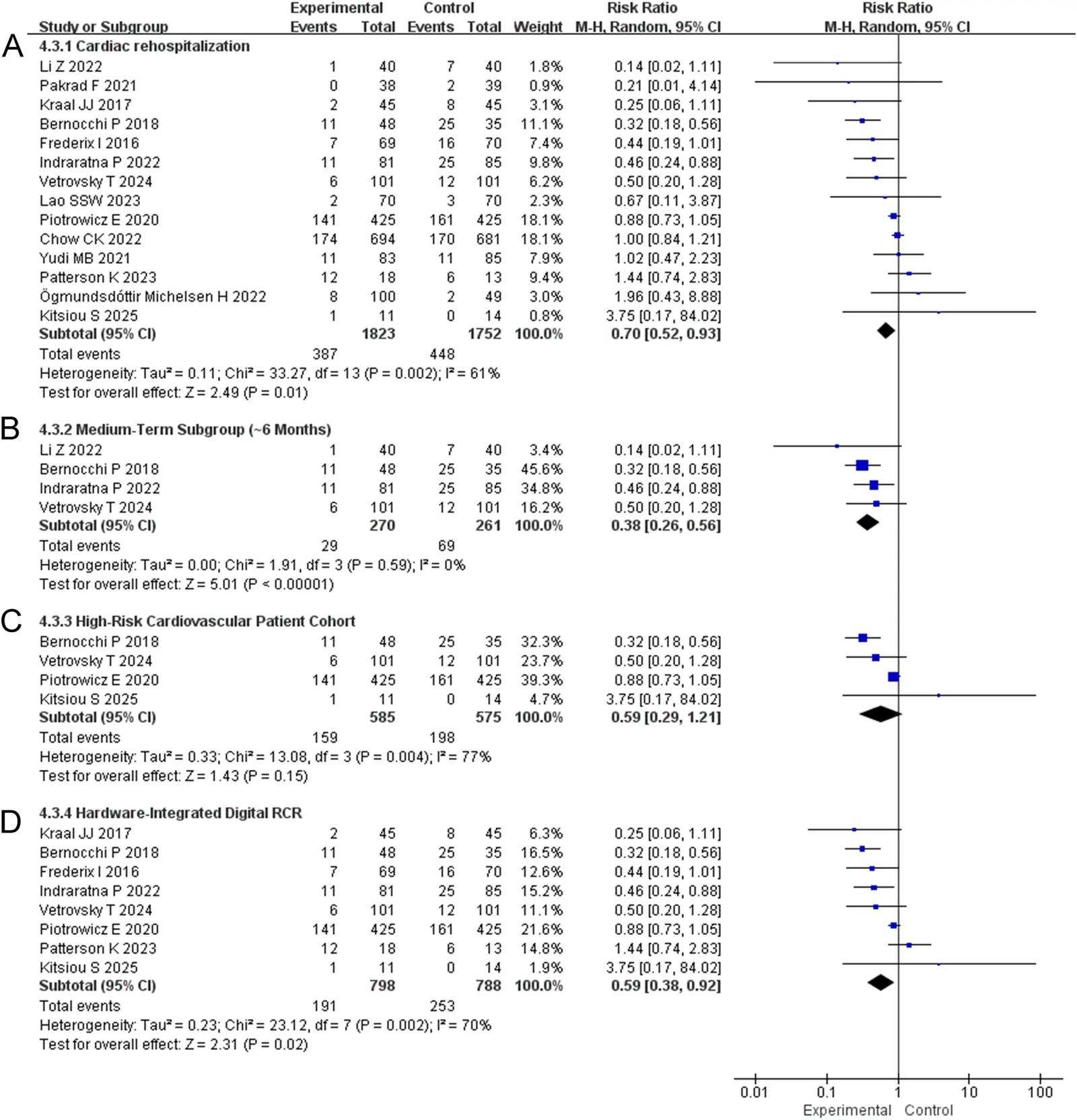
Forest plots of the effects of remote cardiac rehabilitation (RCR) on the risk of cardiac rehospitalization. **(A)** Overall cardiac rehospitalization; **(B)** Medium-term subgroup (∼6 months); **(C)** High-risk cardiovascular patient cohort; **(D)** Hardware-integrated digital RCR modality.

Subgroup analyses stratified by cardiovascular risk profiles, follow-up durations, and intervention modalities were conducted to further elucidate the impact of RCR on cardiac rehospitalization, as shown in Figure 5 and Supplementary Material. Stratification by cardiovascular risk revealed that while the reduction in cardiac hospitalization did not reach formal statistical significance in either subgroup, an exploratory numerical protective trend was observed among high-risk patients compared to low-to-moderate risk cohorts (RR: 0.59 vs 0.74, *P* = 0.15). In terms of follow-up duration, a favorable association was evident within the 6-month observation subgroup (RR = 0.38, *P* < 0.00001) rather than in short-term or long-term windows (RR: 0.94 vs. 0.91, *P* > 0.05). Furthermore, exploratory subgroup analysis showed a statistically significant reduction in cardiac rehospitalization risk within the hardware-integrated digital RCR subgroup (RR = 0.59, *P* = 0.02), whereas the software-only digital RCR subgroup did not reach statistical significance (RR = 0.77, *P* = 0.51; Figures 5B–D).

### Assessment of publication bias, heterogeneity, and trial sequential analysis

Despite the diversity of RCR interventions and assessment scales included in this study, the funnel plots revealed no obvious publication bias (Supplementary Material), and statistical heterogeneity remained predominantly low (*I*^2^< 50%). For outcomes comprising ≥10 studies, quantitative Egger's regression tests confirmed no significant publication bias for cardiac rehospitalization (*P* = 0.133) and all-cause readmission (*P* = 0.874).

To ensure the robustness of conclusions and adhere to methodological guidelines, the meta-analysis applied a random-effects framework for the primary meta-analyses, with fixed-effect models serving as sensitivity analyses (Supplementary Material). Under this dual-model approach demonstrated the stability of our primary outcomes, specifically in the subgroup analyses of HRQoL and cardiac hospitalization. Comprehensive results comparing the estimates from both models were detailed in Supplementary Table.

Trial sequential analysis revealed that the cumulative Z-curve for cardiac rehospitalization crossed both conventional and prespecified monitoring boundaries, whereas that for all-cause readmission touched the conventional threshold but failed to breach the monitoring boundary, as shown in Figures 6A, B. Given that neither analysis reached its required information size (RIS=5615 and RIS=4334, respectively), current evidence remains statistically non-definitive, warranting further adequately powered confirmatory trials.

**Figure 6:**
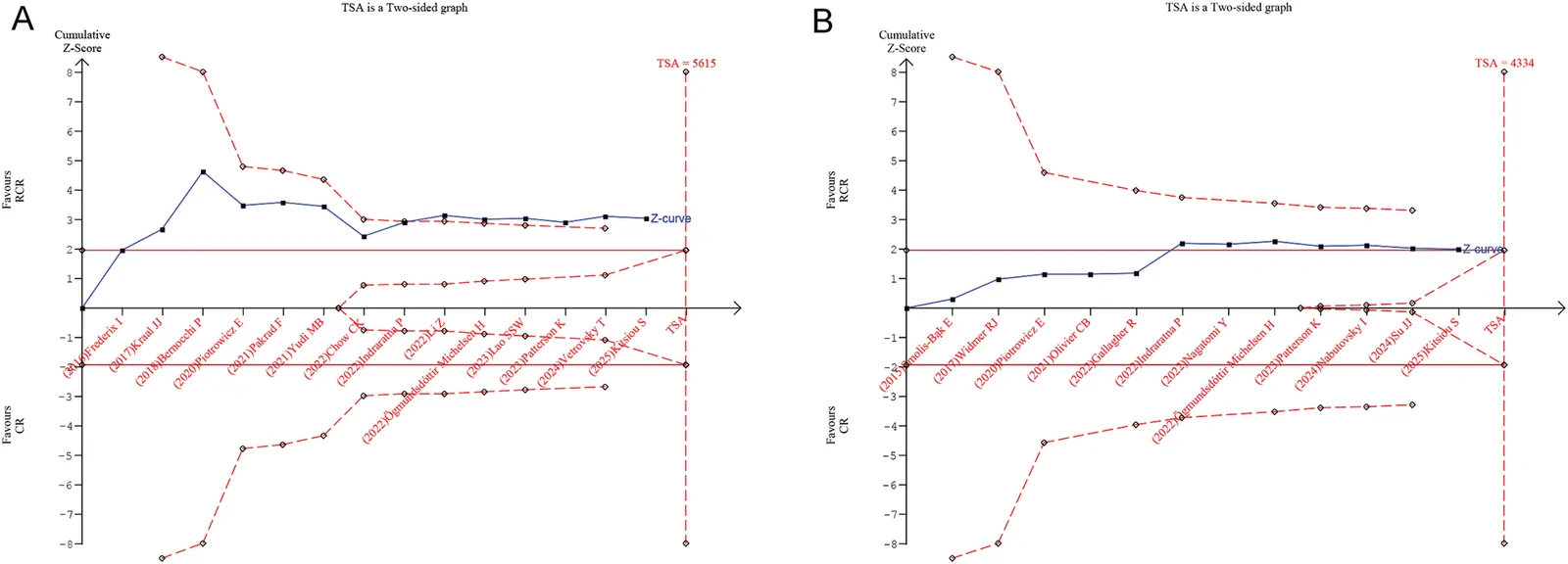
Trial sequential analysis assessing the robustness of evidence for readmission outcomes. **(A)** Cardiac rehospitalization; **(B)** All-cause readmission.

### Health economic outcomes

The health economic outcomes of home-based telerehabilitation vary substantially across different macroeconomic settings, presenting three distinct profiles: dominant (cost-saving), cost-effective, and not cost-effective. First, RCR can manifest as a dominant strategy by simultaneously improving clinical outcomes and lowering total expenditures; as evidenced in Belgium (43), a 24-week program significantly decreased the average medical cost per patient from €2,720 to €2,156 (*P* = 0.01), yielding a highly favorable incremental cost-effectiveness ratio (ICER) of -€21,707/QALY. This cost-saving advantage is further corroborated by evidence from other high-income nations: a real-time videoconferencing rehabilitation study in Australia (44) demonstrated a significant reduction in total healthcare costs by Aus $1,590 per patient, while the Dutch FIT@Home trial (17), adopting a societal perspective, revealed that home-based interventions could save up to €3,160 per capita. Furthermore, these findings align with trends observed in middle-income countries, where the direct implementation costs of home-based programs are substantially lower than those of traditional center-based care ($61 vs. $138) (35).

RCR can increase total expenditures but remain highly cost-effective within standard national willingness-to-pay thresholds; the TELEREH-HF trial (57) demonstrated that while hybrid comprehensive telerehabilitation required an additional cost of 2,581.20 PLN per patient, it yielded an incremental cost-utility ratio (ICUR) of 95,955 PLN/QALY, remaining well below the statutory national threshold of 155,514 PLN/QALY. Third, RCR may fail to establish cost-effectiveness if high setup outlays are not offset by subsequent reductions in healthcare utilization; the ToDo-CR trial (22) incurred a per-patient implementation cost of Aus $1,086.55 and significantly increased 6-month emergency department expenses (Aus $315.83 vs. $136.00; *p* = 0.04), leading to an unacceptably high ICER of Aus $93,395.50 for quality-of-life improvements. The TeleClinical Care pilot (52) required a total implementation outlay of Aus $82,408, translating to a net expenditure of Aus $6,028 per avoided cardiac readmission during the 20-month trial, though long-term projections indicate cost-saving potential only when scaling enrollment beyond 237 patients.

## Discussion

Previous systematic reviews have primarily focus on short-term physiological benefits (58–60), including the six-minute walk distance and peak VO2, alongside implementation-centric indicators like feasibility and patient adherence (5, 61–63). In contrast, this meta-analysis provides comprehensive evidence across long-term HRQoL, cardiovascular safety, and economic viability. Specifically, our study indicates that RCR improves overall HRQoL compared with conventional CR, as reflected in the MacNew and MLHFQ scales, while the generic SF-36 overall score remains comparable between groups. RCR demonstrates a favorable cardiovascular safety profile, with no evidence of increased risk for MACEs, all-cause mortality, or adverse events compared with conventional CR, while significantly reducing the risk of cardiac rehospitalization. These findings establish RCR not merely as a feasible alternative to conventional CR, but as an effective strategy for cardiac functional recovery.

This study used four distinct HRQoL scales to evaluate physical recovery and psychological well-being, providing a more precise assessment of patient-centered rehabilitation outcomes than single-scale assessments (64, 65). The multidimensional approach indicated that RCR was associated with improvements in selected HRQoL domains, particularly physical functioning, while evidence for psychological benefits was less consistent. The physical improvements were observed beyond 12 months across both SF-36 and MacNew scales, reflecting the cumulative benefits of remote exercise adherence and habit formation (66, 67). Conversely, psychological outcomes exhibited mixed findings: although short-term improvement were captured on the SF-36 mental scale at 6 months, other validated tools such as the HADS demonstrated comparable levels between RCR and center-based CR. The psychological responsiveness to digital interventions may vary substantially depending on measurement tools and baseline distress (68–70). This psychological improvement holds substantial prognostic value, considering that depression and anxiety act as independent drivers of adverse cardiovascular events (71, 72). These findings highlight the potential value of a stepped-care RCR model (73), transitioning from early psychological support to long-term digital maintenance for sustained self-management and physical functional recovery.

Subgroup analyses of cardiovascular safety revealed an exploratory numerical protective trend in cardiac hospitalization among high-risk patients, with statistically significant favorable associations observed within the 6-month observation subgroup and the hardware-integrated digital RCR modality. The inherently low event rates in low-to-intermediate-risk cohorts create a statistical ‘floor effect’ that masks therapeutic gains (74–76), whereas high-risk patients offer a significantly larger margin for clinical optimization. However, these exploratory estimates did not reach statistical significance and cannot establish high-risk patients as preferential beneficiaries. Rather than establishing 6 months as an intrinsically optimal therapeutic window, the marked risk reduction observed in the 6-month subgroup represents an empirical association that may reflect trial-level characteristics and study distribution across follow-up intervals. The statistically significant association observed within the hardware-integrated digital RCR subgroup provides explicitly hypothesis-generating evidence, which may be attributed to continuous physiological telemonitoring that enables prompt adaptations to exercise regimens. This real-time data allows clinicians to deliver timely medical interventions and dynamic prescription updates, thereby reinforcing proactive secondary prevention. These subgroup findings should be interpreted cautiously and considered hypothesis-generating, warranting future head-to-head trials to evaluate structured, hardware-enabled interventions across specific cardiovascular risk strata.

Unlike previous purely clinical-centric reviews (58, 59), this study extends the evaluation to include a health-economic perspective. The potential economic feasibility of RCR is largely driven by its ability to reduce costly all-cause readmissions (RR = 0.91) and cardiac rehospitalizations (RR = 0.70) (77). While remote rehabilitation offers the dual benefits of conserving medical resources and minimizing patients’ commuting costs (78), its overall economic viability remains uncertain, critically depending on initial setup outlays, patient scaling, and subsequent healthcare utilization. Although establishing remote monitoring platforms requires initial infrastructure investments, economies of scale suggest that the marginal cost per patient may decrease as implementation expands. However, given that current evidence predominantly stems from small-scale clinical trials, the optimal cost-benefit ratio for real-world deployment remains undetermined, particularly across varying national economic levels. These health-economic outcomes, however, depend heavily on the macro-economic context. While high-income nations may leverage RCR primarily to curtail long-term secondary prevention costs within mature digital infrastructures, low- and middle-income countries (LMICs) might explore it to bridge the geographical disparity in healthcare delivery caused by urban centralization. Despite non-negligible upfront infrastructure costs for LMICs, long-term RCR implementation could potentially offer a scalable solution to the chronic shortage of specialized cardiac rehabilitation facilities and personnel in rural areas.

Although statistical heterogeneity remained low across our analyses, we acknowledge inherent clinical diversity within the pooled cohorts, as our predefined high-risk subgroup represents a heterogeneous composite population. While our analytical framework employed a dual-model approach—with the random-effects model specifically accounting for between-study variability—it cannot eliminate clinical heterogeneity or establish uniform treatment responsiveness across different clinical phenotypes. The true magnitude of RCR benefit may differ substantially among individual high-risk phenotypes. TSA indicated that the cumulative Z-curve for cardiac rehospitalization crossed the prespecified monitoring boundary without reaching the required information size (RIS=5615). Consequently, as current evidence remains statistically non-definitive, subsequent adequately powered multicenter trials remain necessary to confirm treatment protocols.

In summary, RCR delivers physical and psychological therapeutic effects comparable to conventional center-based programs with a favorable cardiovascular safety profile. Notably, exploratory subgroup analyses indicate that the reduction in cardiac rehospitalization was primarily observed in hardware-integrated interventions and over a 6-month follow-up period, accompanied by an exploratory numerical protective trend in high-risk patients. However, these observational associations should be considered hypothesis-generating in the absence of definitive subgroup interaction. These results provide a preliminary evidence base to support the integration of RCR into clinical practice and health-policy frameworks. By integrating these clinical and economic advantages, RCR successfully overcomes traditional access barriers, thereby substantially expanding the global coverage of cardiac rehabilitation. Future research should prioritize large-scale, long-term, multicenter randomized controlled trials, with a specific focus on including high-risk patient cohorts, such as those with severe heart failure or complex coronary artery disease. To fully realize the clinical and economic potential of RCR, future implementation should transition toward a standardized yet personalized stepped-care model (73). This involves front-loading intensive psychological and physical support during the early post-discharge window and subsequently tapering to low-intensity remote rehabilitation support (73, 79), allowing clinicians and policymakers to translate the potential economic and clinical advantages of RCR into long-term patient recovery and health-system sustainability.

## Limitations

The inherent heterogeneity across included studies warrants cautious interpretation. Given that the high-risk subgroup represents a clinically heterogeneous composite population combining distinct phenotypes, the pooled estimates may not reflect uniform efficacy across all specific high-risk phenotypes. Most included trials were single-center studies with modest sample sizes and follow-up largely restricted to ≤ 12 months, constraining the generalizability of our findings regarding long-term durability and safety in advanced cardiovascular cohorts.

## Conclusions

RCR may serve as an effective and safe alternative to conventional care that improves physical and psychological well-being, was associated with a lower risk of cardiac rehospitalization, and may provide economic value in certain healthcare systems. Despite protective associations observed across hardware-integrated modalities, the 6-month observation subgroup, and high-risk patients, current evidence is insufficient to establish preferential subgroup efficacy, warranting dedicated confirmatory trials.

## Data Availability

The original contributions presented in the study are included in the article/Supplementary Material, further inquiries can be directed to the corresponding authors.

## References

[1] Rao SV O’Donoghue ML Ruel M Rab T Tamis-Holland JE Alexander JH. 2025 ACC/AHA/ACEP/NAEMSP/SCAI guideline for the management of patients with acute coronary syndromes: a report of the American College of Cardiology/American Heart Association Joint Committee on Clinical Practice Guidelines. Circulation. (2025) 151(13):e771–e862. 10.1161/CIR.0000000000001309 40014670

[2] Ilonze OJ Forman DE LeMond L Myers J Hummel S Vest AR. Beyond guideline-directed medical therapy: nonpharmacologic management for patients with heart failure. JACC Heart Fail. (2025) 13(2):185–99. 10.1016/j.jchf.2024.08.018 39453358

[3] Martin SS Aday AW Allen NB Almarzooq ZI Anderson CAM Arora P. 2025 Heart disease and stroke statistics: a report of US and global data from the American Heart Association. Circulation. (2025) 151(8):e41–e660. 10.1161/CIR.0000000000001303 39866113 PMC12256702

[4] Palaniappan LP Allen NB Almarzooq ZI Anderson CAM Arora P Avery CL. 2026 Heart disease and stroke statistics: a report of US and global data from the American Heart Association. Circulation. (2026) 153(9):e275–e906. 10.1161/CIR.0000000000001412 41562125

[5] Li R Wang M Chen S Zhang L. Comparative efficacy and adherence of telehealth cardiac rehabilitation interventions for patients with cardiovascular disease: a systematic review and network meta-analysis. Int J Nurs Stud. (2024) 158:104845. 10.1016/j.ijnurstu.2024.104845 39032245

[6] Li S Xu H. The development of cardiac rehabilitation in China: current status and future perspectives. Rev Cardiovasc Med. (2024) 25(7):233. 10.31083/j.rcm2507233 39139407 PMC11317330

[7] Klein A Pinheiro RF Fonseca-Pinto R. Integrating internet of things into cardiac rehabilitation for heart failure: a review of emerging technologies. Front Med (Lausanne). (2025) 12:1737523. 10.3389/fmed.2025.1737523 41657572 PMC12879099

[8] Scherrenberg M Falter M Abreu A Aktaa S Busnatu S Casado-Arroyo R. Standards for cardiac telerehabilitation. Eur Heart J. (2025) 46(38):3714–37. 10.1093/eurheartj/ehaf408 40742158

[9] Piotrowicz E Pencina MJ Opolski G Zareba W Banach M Kowalik I. Effects of a 9-week hybrid comprehensive telerehabilitation program on long-term outcomes in patients with heart failure: the telerehabilitation in heart failure patients (TELEREH-HF) randomized clinical trial. JAMA Cardiol. (2020) 5(3):300–8. 10.1001/jamacardio.2019.5006 31734701 PMC6865325

[10] Li L Ringeval M Wagner G Pare G Ozemek C Kitsiou S. Effectiveness of home-based cardiac rehabilitation interventions delivered via Mhealth technologies: a systematic review and meta-analysis. Lancet Digit Health. (2025) 7(4):e238–e54. 10.1016/j.landig.2025.01.011 40023729

[11] Bae J-W Woo S-I Lee J Park S-D Kwon SW Choi SH. mHealth interventions for lifestyle and risk factor modification in coronary heart disease: randomized controlled trial. JMIR Mhealth Uhealth. (2021) 9(9):e29928. 10.2196/29928 34559058 PMC8501404

[12] Batalik L Pepera G Papathanasiou J Rutkowski S Líška D Batalikova K. Is the training intensity in phase two cardiovascular rehabilitation different in telehealth versus outpatient rehabilitation? J Clin Med. (2021) 10(18):4069. 10.3390/jcm10184069 34575185 PMC8466823

[13] Chow CK Klimis H Thiagalingam A Redfern J Hillis GS Brieger D. Text messages to improve medication adherence and secondary prevention after acute coronary syndrome: the TEXTMEDS randomized clinical trial. Circulation. (2022) 145(19):1443–55. 10.1161/CIRCULATIONAHA.121.056161 35533220

[14] Claes J Cornelissen V McDermott C Moyna N Pattyn N Cornelis N. Feasibility, acceptability, and clinical effectiveness of a technology-enabled cardiac rehabilitation platform (physical activity toward health-I): randomized controlled trial. J Med Internet Res. (2020) 22(2):e14221. 10.2196/14221 32014842 PMC7055834

[15] Gallagher R Chow CK Parker H Neubeck L Celermajer DS Redfern J. The effect of a game-based mobile app ‘myheartmate’ to promote lifestyle change in coronary disease patients: a randomized controlled trial. Eur Heart J Digit Health. (2023) 4(1):33–42. 10.1093/ehjdh/ztac069 36743873 PMC9890079

[16] Kamel H Hafez MS Bastawy I. Telemedicine improves the short-term medical care of acute ST-segment elevation myocardial infarction after primary percutaneous coronary intervention. Front Cardiovasc Med. (2021) 8:693731. 10.3389/fcvm.2021.693731 34322529 PMC8311002

[17] Kraal JJ Van den Akker-Van Marle ME Abu-Hanna A Stut W Peek N Kemps HM. Clinical and cost-effectiveness of home-based cardiac rehabilitation compared to conventional, centre-based cardiac rehabilitation: results of the FIT@Home study. Eur J Prev Cardiol. (2017) 24(12):1260–73. 10.1177/2047487317710803 28534417 PMC5518918

[18] Lao SSW Chair SY Wang Q Leong MLT. The feasibility and effects of smartphone-based application on cardiac rehabilitation for patients after percutaneous coronary intervention: a randomized controlled trial. J Cardiovasc Nurs. (2024) 39(1):88–101. 10.1097/JCN.0000000000000993 37088896

[19] Li X Zhao L Xu T Shi G Li J Shuai W. Cardiac telerehabilitation under 5g internet of things monitoring: a randomized pilot study. Sci Rep. (2023) 13(1):18886. 10.1038/s41598-023-46175-z 37919385 PMC10622509

[20] Ögmundsdóttir Michelsen H Sjölin I Bäck M Gonzalez Garcia M Olsson A Sandberg C. Effect of a lifestyle-focused web-based application on risk factor management in patients who have had a myocardial infarction: randomized controlled trial. J Med Internet Res. (2022) 24(3):e25224. 10.2196/25224 PMC9015765 35357316

[21] Pakrad F Ahmadi F Grace SL Oshvandi K Kazemnejad A. Traditional vs extended hybrid cardiac rehabilitation based on the continuous care model for patients who have undergone coronary artery bypass surgery in a middle-income country: a randomized controlled trial. Arch Phys Med Rehabil. (2021) 102(11):2091–101 e3. 10.1016/j.apmr.2021.04.026 34175270

[22] Patterson K Davey R Keegan R Niyonsenga T Mohanty I Bowen S. Testing the effect of a smartphone app on hospital admissions and sedentary behavior in cardiac rehabilitation participants: ToDo-CR randomized controlled trial. JMIR Mhealth Uhealth. (2023) 11:e48229. 10.2196/48229 37788043 PMC10582808

[23] Pfaeffli Dale L Whittaker R Jiang Y Stewart R Rolleston A Maddison R. Text message and internet support for coronary heart disease self-management: results from the Text4Heart randomized controlled trial. J Med Internet Res. (2015) 17(10):e237. 10.2196/jmir.4944 26490012 PMC4642389

[24] Seron P Oliveros MJ Marzuca-Nassr GN Morales G Román C Muñoz SR. Hybrid cardiac rehabilitation program in a low-resource setting: a randomized clinical trial. JAMA Netw Open. (2024) 7(1):e2350301. 10.1001/jamanetworkopen.2023.50301 38194236 PMC10777264

[25] Skobel E Knackstedt C Martinez-Romero A Salvi D Vera-Munoz C Napp A. Internet-based training of coronary artery patients: the heart cycle trial. Heart Vessels. (2017) 32(4):408–18. 10.1007/s00380-016-0897-8 27730298

[26] Snoek JA Prescott EI Van Der Velde AE Eijsvogels TMH Mikkelsen N Prins LF. Effectiveness of home-based mobile guided cardiac rehabilitation as alternative strategy for nonparticipation in clinic-based cardiac rehabilitation among elderly patients in Europe: a randomized clinical trial. JAMA Cardiol. (2021) 6(4):463–8. 10.1001/jamacardio.2020.5218 33112363 PMC7593879

[27] Taylor JL Holland DJ Mielke GI Bailey TG Johnson NA Leveritt MD. Effect of high-intensity interval training on visceral and liver fat in cardiac rehabilitation: a randomized controlled trial. Obesity (Silver Spring). (2020) 28(7):1245–53. 10.1002/oby.22833 32475048

[28] Sunamura M Ter Hoeve N Van Den Berg-Emons RJG Geleijnse ML Haverkamp M Stam HJ. Randomised controlled trial of two advanced and extended cardiac rehabilitation programmes. Heart. (2018) 104(5):430–7. 10.1136/heartjnl-2017-311681 28954826

[29] Widmer RJ Allison TG Lennon R Lopez-Jimenez F Lerman LO Lerman A. Digital health intervention during cardiac rehabilitation: a randomized controlled trial. Am Heart J. (2017) 188:65–72. 10.1016/j.ahj.2017.02.016 28577682

[30] Yudi MB Clark DJ Tsang D Jelinek M Kalten K Joshi SB. Smartphone-based, early cardiac rehabilitation in patients with acute coronary syndromes: a randomized controlled trial. Coron Artery Dis. (2021) 32(5):432–40. 10.1097/MCA.0000000000000938 32868661

[31] Li Z Hui Z Zheng Y Yu J Zhang J. Efficacy of phase ii remote home rehabilitation in patients with acute myocardial infarction after percutaneous coronary intervention. Contrast Media Mol Imaging. (2022) 2022:4634769. 10.1155/2022/4634769 35815057 PMC9208999

[32] Nagatomi Y Ide T Higuchi T Nezu T Fujino T Tohyama T. Home-based cardiac rehabilitation using information and communication technology for heart failure patients with frailty. ESC Heart Fail. (2022) 9(4):2407–18. 10.1002/ehf2.13934 35534907 PMC9288767

[33] Olivier CB Middleton SK Purington N Shashidhar S Hereford J Mahaffey KW. Why digital health trials can fail: lessons learned from a randomized trial of health coaching and virtual cardiac rehabilitation. Cardiovasc Digit Health J. (2021) 2(2):101–8. 10.1016/j.cvdhj.2021.01.003 35265897 PMC8890340

[34] Piotrowicz E Baranowski R Bilinska M Stepnowska M Piotrowska M Wójcik A. A new model of home-based telemonitored cardiac rehabilitation in patients with heart failure: effectiveness, quality of life, and adherence. Eur J Heart Fail. (2010) 12(2):164–71. 10.1093/eurjhf/hfp181 20042423

[35] De Lima AP Pereira DG Nascimento IO Martins TH Oliveira AC Nogueira TS. Cardiac telerehabilitation in a middle-income country: analysis of adherence, effectiveness and cost through a randomized clinical trial. Eur J Phys Rehabil Med. (2022) 58(4):598–605. 10.23736/S1973-9087.22.07340-3 35634888 PMC9980526

[36] Devi R Powell J Singh S. A web-based program improves physical activity outcomes in a primary care angina population: randomized controlled trial. J Med Internet Res. (2014) 16(9):e186. 10.2196/jmir.3340 25217464 PMC4180351

[37] Hawkes AL Patrao TA Atherton J Ware RS Taylor CB O’Neil A. Effect of a telephone-delivered coronary heart disease secondary prevention program (proactive heart) on quality of life and health behaviours: primary outcomes of a randomised controlled trial. Int J Behav Med. (2013) 20(3):413–24. 10.1007/s12529-012-9250-5 23012159

[38] Leemrijse CJ Peters RJG Von Birgelen C Van Dijk L Van Hal JMC Kuijper AFM. The telephone lifestyle intervention ‘hartcoach’ has modest impact on coronary risk factors: a randomised multicentre trial. Eur J Prev Cardiol. (2016) 23(15):1658–68. 10.1177/2047487316639681 27000097

[39] Pinto BM Dunsiger SI Farrell N Marcus BH Todaro JF. Psychosocial outcomes of an exercise maintenance intervention after phase ii cardiac rehabilitation. J Cardiopulm Rehabil Prev. (2013) 33(2):91–8. 10.1097/HCR.0b013e3182825531 23422351 PMC3586302

[40] Piotrowicz E Zieliński T Bodalski R Rywik T Dobraszkiewicz-Wasilewska B Sobieszczańska-Małek M. Home-based telemonitored nordic walking training is well accepted, safe, effective and has high adherence among heart failure patients, including those with cardiovascular implantable electronic devices: a randomised controlled study. Eur J Prev Cardiol. (2015) 22(11):1368–77. 10.1177/2047487314551537 25261268

[41] Su JJ Yu DS. Effects of a nurse-led eHealth cardiac rehabilitation programme on health outcomes of patients with coronary heart disease: a randomised controlled trial. Int J Nurs Stud. (2021) 122:104040. 10.1016/j.ijnurstu.2021.104040 34333211

[42] Bravo-Escobar R González-Represas A Gómez-González AM Montiel-Trujillo A Aguilar-Jimenez R Carrasco-Ruíz R. Effectiveness and safety of a home-based cardiac rehabilitation programme of mixed surveillance in patients with ischemic heart disease at moderate cardiovascular risk: a randomised, controlled clinical trial. BMC Cardiovasc Disord. (2017) 17(1):66. 10.1186/s12872-017-0499-0 28219338 PMC5319164

[43] Frederix I Hansen D Coninx K Vandervoort P Vandijck D Hens N. Effect of comprehensive cardiac telerehabilitation on one-year cardiovascular rehospitalization rate, medical costs and quality of life: a cost-effectiveness analysis. Eur J Prev Cardiol. (2016) 23(7):674–82. 10.1177/2047487315602257 26289723

[44] Hwang R Morris NR Mandrusiak A Bruning J Peters R Korczyk D. Cost-utility analysis of home-based telerehabilitation compared with centre-based rehabilitation in patients with heart failure. Heart Lung Circ. (2019) 28(12):1795–803. 10.1016/j.hlc.2018.11.010 30528811

[45] Peng X Su Y Hu Z Sun X Li X Dolansky MA. Home-based telehealth exercise training program in Chinese patients with heart failure: a randomized controlled trial. Medicine (Baltimore). (2018) 97(35):e12069. 10.1097/MD.0000000000012069 30170422 PMC6392598

[46] Schmidt C Magalhães S Gois Basilio P Santos C Oliveira MI Ferreira JP. Center- vs home-based cardiac rehabilitation in patients with heart failure: EXIT-HF randomized controlled trial. JACC Heart Fail. (2025) 13(5):695–706. 10.1016/j.jchf.2024.09.024 39895436

[47] Bernocchi P Vitacca M La Rovere MT Volterrani M Galli T Baratti D. Home-based telerehabilitation in older patients with chronic obstructive pulmonary disease and heart failure: a randomised controlled trial. Age Ageing. (2018) 47(1):82–8. 10.1093/ageing/afx146 28985325

[48] Lundgren K Langlo K Salvesen Ø Aspvik N Mo R Ellingsen Ø. Exercise-based telerehabilitation for heart failure patients declining outpatient rehabilitation-a randomized controlled trial. Med Sci Sports Exerc. (2025) 57(3):449–60. 10.1249/MSS.0000000000003590 39501477

[49] Herring LY Dallosso H Schreder S Smith EJ Waheed G Gray LJ. Physical activity after cardiac events (PACES): a group education programme with subsequent text message support designed to increase physical activity in individuals with diagnosed coronary heart disease: a randomised controlled trial. Open Heart. (2021) 8(1):e001351. 10.1136/openhrt-2020-001351 33637567 PMC7919588

[50] Maddison R Pfaeffli L Whittaker R Stewart R Kerr A Jiang Y. A mobile phone intervention increases physical activity in people with cardiovascular disease: results from the heart randomized controlled trial. Eur J Prev Cardiol. (2015) 22(6):701–9. 10.1177/2047487314535076 24817694

[51] Vetrovsky T Siranec M Frybova T Gant I Svobodova I Linhart A. Lifestyle walking intervention for patients with heart failure with reduced ejection fraction: the watchful trial. Circulation. (2024) 149(3):177–88. 10.1161/CIRCULATIONAHA.123.067395 37955615 PMC10782943

[52] Indraratna P Biswas U McVeigh J Mamo A Magdy J Vickers D. A smartphone-based model of care to support patients with cardiac disease transitioning from hospital to the community (Teleclinical Care): pilot randomized controlled trial. JMIR Mhealth Uhealth. (2022) 10(2):e32554. 10.2196/32554 35225819 PMC8922139

[53] Smolis-Bąk E Dąbrowski R Piotrowicz E Chwyczko T Dobraszkiewicz-Wasilewska B Kowalik I. Hospital-based and telemonitoring guided home-based training programs: effects on exercise tolerance and quality of life in patients with heart failure (NYHA class iii) and cardiac resynchronization therapy. A randomized, prospective observation. Int J Cardiol. (2015) 199:442–7. 10.1016/j.ijcard.2015.07.041 26276068

[54] Kitsiou S Gerber BS Buchholz SW Kansal MM Sun J Pressler SJ. Patient-centered mHealth intervention to improve self-care in patients with chronic heart failure: phase 1 randomized controlled trial. J Med Internet Res. (2025) 27:e55586. 10.2196/55586 39813671 PMC11780297

[55] Nabutovsky I Breitner D Heller A Levine Y Moreno M Scheinowitz M. Home-based cardiac rehabilitation among patients unwilling to participate in hospital-based programs. J Cardiopulm Rehabil Prev. (2024) 44(1):33–9. 10.1097/HCR.0000000000000796 37220026

[56] Hwang R Bruning J Morris NR Mandrusiak A Russell T. Home-based telerehabilitation is not inferior to a centre-based program in patients with chronic heart failure: a randomised trial. J Physiother. (2017) 63(2):101–7. 10.1016/j.jphys.2017.02.017 28336297

[57] Niewada M Tabor B Piotrowicz E Piotrowicz R Opolski G Banach M. Cost-effectiveness of telerehabilitation in patients with heart failure in Poland: an analysis based on the results of telerehabilitation in the heart failure patients (TELEREH-HF) randomized clinical trial. Kardiol Pol. (2021) 79(5):510–6. 10.33963/KP.15885 34125923

[58] Tang J Ma D Li M Ding Y Zhou X He H. Outcomes of electronic health interventions for patients with heart failure: a network meta-analysis. Herz. (2025) 50(4):268–76. 10.1007/s00059-025-05301-5 40327069

[59] De Lathauwer ILJ Nieuwenhuys WW Hafkamp F Regis M Brouwers RWM Funk M. Remote patient monitoring in heart failure: a comprehensive meta-analysis of effective programme components for hospitalization and mortality reduction. Eur J Heart Fail. (2025) 27(9):1670–85. 10.1002/ejhf.3568 39834044 PMC12502459

[60] Alqahtani AS. Telehealth-based cardiac rehabilitation for heart failure: a systematic review of effectiveness, access, and patient-centred outcome. Medicina (Kaunas). (2026) 62(1):25. 10.3390/medicina62010025 PMC12842665 41597311

[61] Coorey GM Neubeck L Mulley J Redfern J. Effectiveness, acceptability and usefulness of mobile applications for cardiovascular disease self-management: systematic review with meta-synthesis of quantitative and qualitative data. Eur J Prev Cardiol. (2018) 25(5):505–21. 10.1177/2047487317750913 29313363

[62] Deckwart O Koehler K Lezius S Prescher S Koehler F Winkler S. Effects of remote patient management on self-care behaviour in heart failure patients: results from the randomized TIM-HF2 trial. Eur J Cardiovasc Nurs. (2023) 22(8):786–94. 10.1093/eurjcn/zvad019 36752782

[63] Oszczygiel M Völler H Schröder K Popescu-Schuh I-S Schromm E Heinze V. Adherence to drug therapy in patients with heart failure associated with reduced ejection fractions (HFrEF) during cardiac rehabilitation and after 3 and 6 months. Clin Res Cardiol. (2024) 113(6):866–74. 10.1007/s00392-023-02314-0 37796317

[64] Fallah Z Feizi A Sadeghi M Hadavi MM Shahnazi H. The effect of home-based virtual exercise rehabilitation on myocardial infarction patients using the health action process approach: a randomized controlled trial. BMC Sports Sci Med Rehabil. (2026) 18(1):35. 10.1186/s13102-025-01492-w PMC12837327 41430286

[65] Shi Y Xu H Dong J. Exercise-based cardiac rehabilitation for patients undergoing coronary artery operation: a systematic review and meta-analysis based on current randomized controlled trials. Int J Surg. (2025) 111(3):2708–21. 10.1097/JS9.0000000000002268 39903572 PMC12372725

[66] Sari C Heesch CM Kovacs JA Simon A Andreka P. Participation in a comprehensive cardiac rehabilitation program improves mid- and long-term prognosis in survivors of acute coronary syndrome. Am J Prev Cardiol. (2025) 23:101042. 10.1016/j.ajpc.2025.101042 40606515 PMC12221284

[67] Breeman LD Janssen VR Kraaijenhagen RA Al-Dhahir I IJzerman RVH Wolstencroft K. Lifestyle behaviour change of patients following cardiac rehabilitation: the benefit intervention study with one-year follow-up. Eur J Prev Cardiol. (2025). 10.1093/eurjpc/zwaf193 40203287

[68] Wells A Reeves D Heal C Fisher P Doherty P Davies L. Metacognitive therapy home-based self-help for anxiety and depression in cardiovascular disease patients in the UK: a single-blind randomised controlled trial. PLoS Med. (2023) 20(1):e1004161. 10.1371/journal.pmed.1004161 36719886 PMC9888717

[69] Liu Y Wang X Li H Wang C Kong M Zhang M. The impact of home-based telerehabilitation versus center-based cardiac rehabilitation on anxiety, depression, and quality of life after coronary artery bypass grafting: a randomized controlled trial. BMC Cardiovasc Disord. (2026) 26(1):499. 10.1186/s12872-026-05703-1 42035003 PMC13262403

[70] Yang X Liang J Li Y Miao J Xu Y Li W. Comparison of mHealth-supported and centre-based cardiac rehabilitation in patients with stable coronary heart disease: a randomized controlled trial. BMC Cardiovasc Disord. (2026) 26(1):610. 10.1186/s12872-026-05991-7 42177443 PMC13383499

[71] Blatch Armon D Buhayer A Miteva K Meinlschmidt G Dobretz K Zuccarella-Hackl C. Depression and cardiovascular disease: mind the gap in the guidelines. Eur Heart J. (2025) 46(41):4226–69. 10.1093/eurheartj/ehaf479 40878995 PMC12579981

[72] Douma ER Kop WJ Kupper N. Associations between psychological factors and adherence to health behaviors after percutaneous coronary intervention: the role of cardiac rehabilitation. Ann Behav Med. (2024) 58(5):328–40. 10.1093/abm/kaae008 38431284 PMC11008588

[73] Reed JL Mullen K-A Small S Moran C Mir H Tulloch H. An ounce of prevention and a pound of cardiac rehabilitation. Can J Cardiol. (2026) 42(5S):S22–S34. 10.1016/j.cjca.2026.02.036 42161478

[74] Correia VM Morales Ribeiro V Henkes Machado R Defante MLR De Souza K De Oliveira Silva Filho JR. Exercise training in hypertrophic cardiomyopathy: a meta-analysis of randomised trials with trial sequential analysis. Heart. (2025). 10.1136/heartjnl-2025-326377 41320293

[75] Szalewska D Główczyńska R Piotrowicz R Kowalik I Pencina MJ Opolski G. An aetiology-based subanalysis of the telerehabilitation in heart failure patients (TELEREH-HF) trial. ESC Heart Fail. (2021) 8(2):1263–73. 10.1002/ehf2.13189 33527740 PMC8006702

[76] Sadowski K Piotrowicz R Kłopotowski M Wolszakiewicz J Kowalik I Mierzyńska A. Efficacy and safety of hybrid cardiac telerehabilitation in patients with hypertrophic cardiomyopathy without left ventricular outflow tract obstruction and preserved ejection fraction: a randomized clinical trial. Kardiol Pol. (2026) 84(1):66–74. 10.33963/v.phj.110581 41502401

[77] Shah ND Banta CW Berger AL Hattenberger A Zimmerman A Martin BE. Retrospective comparison of outcomes and cost of virtual versus center-based cardiac rehabilitation programs. J Am Heart Assoc. (2024) 13(24):e036861. 10.1161/JAHA.124.036861 39673292 PMC11935551

[78] Walli-Attaei M Halle M Mueller S. Cost-effectiveness of digitally enabled cardiac rehabilitation: progress, promise, and persisting questions. Eur J Prev Cardiol. (2025). 10.1093/eurjpc/zwaf660 41092454

[79] Salah H Ambrosy A Biegus J Bocchi E Butler J Chioncel O. Optimizing pre-to-post discharge transition of care in patients hospitalized for heart failure - part 3 of the international expert opinion series on acute heart failure management. J Card Fail. (2025). 10.1016/j.cardfail.2025.09.020 41027508

