# Supplementary material for: Long-term quality of life, rehospitalization risks, and economic viability of remote cardiac rehabilitation: an updated systematic review and meta-analysis
Source: Front Digit Health. 2026 Oct 14;8:1935832. doi: 10.3389/fdgth.2026.1935832 (PMC13713463; doi:10.3389/fdgth.2026.1935832)
Supplement: Supplementary file 1 [file Datasheet1.docx]

**Supplementary Section 1:**

Detailed literature search strategy for the systematic review and meta-analysis.

**Pubmed Search Strategy**

("Telephone"[Mesh] OR "telephone"[tiab] OR "Cell Phone"[Mesh] OR "cell phone"[tiab] OR "Smartphone"[Mesh] OR "smartphone"[tiab] OR "Text Messaging"[Mesh] OR "text messaging"[tiab] OR "message"[tiab] OR "Internet"[Mesh] OR "internet"[tiab] OR "Online Social Networking"[Mesh] OR "online social networking"[tiab] OR "Mobile Applications"[Mesh] OR "mobile applications"[tiab] OR "Remote Consultation"[Mesh] OR "remote consultation"[tiab] OR "Distance Counseling"[Mesh] OR "distance counseling"[tiab] OR "Telemedicine"[Mesh] OR "telemedicine"[tiab]) AND ("Cardiovascular Diseases"[Mesh] OR "Heart Diseases"[Mesh] OR "Myocardial Ischemia"[Mesh] OR "Coronary Artery Disease"[Mesh] OR "Acute Coronary Syndrome"[Mesh] OR "Myocardial Infarction"[Mesh] OR "Angina Pectoris"[Mesh] OR "Percutaneous Coronary Intervention"[Mesh] OR "Coronary Artery Bypass"[Mesh] OR "Heart Failure"[Mesh] OR "cardiovascular disease*"[tiab] OR "coronary heart disease*"[tiab] OR "coronary artery disease*"[tiab] OR "acute coronary syndrome*"[tiab] OR "myocardial infarction*"[tiab] OR "post-infarction"[tiab] OR "percutaneous coronary intervention*"[tiab] OR "PCI"[tiab] OR "coronary artery bypass*"[tiab] OR "CABG"[tiab] OR "heart failure"[tiab] OR "cardiac failure"[tiab] OR "angina"[tiab]) AND ("Cardiac Rehabilitation"[Mesh] OR "cardiac rehabilitation"[tiab] OR "heart rehabilitation"[tiab] OR "cardiovascular rehabilitation"[tiab] OR "cardiac rehab"[tiab] OR "heart rehab"[tiab] OR "telerehabilitation"[tiab] OR "home-based rehabilitation"[tiab]) Filters: Randomized Controlled Trial

**Embase Search Strategy**

('telephone'/exp OR 'telephone' OR 'telephone':ti,ab OR 'mobile phone'/exp OR 'mobile phone' OR 'mobile phone':ti,ab OR 'smartphone'/exp OR 'smartphone' OR 'smartphone':ti,ab OR 'text messaging'/exp OR 'text messaging' OR 'text messaging':ti,ab OR 'message':ti,ab OR 'internet'/exp OR 'internet' OR 'internet':ti,ab OR 'online social networking'/exp OR 'online social networking' OR 'social media':ti,ab OR 'mobile application'/exp OR 'mobile application' OR 'mobile app':ti,ab OR 'teleconsultation'/exp OR 'teleconsultation' OR 'remote consultation':ti,ab OR 'telemedicine'/exp OR 'telemedicine' OR 'telemedicine':ti,ab) AND ('cardiovascular disease'/exp OR 'cardiovascular disease' OR 'coronary artery disease'/exp OR 'coronary artery disease' OR 'ischemic heart disease'/exp OR 'ischemic heart disease' OR 'heart infarction'/exp OR 'heart infarction' OR 'percutaneous coronary intervention'/exp OR 'percutaneous coronary intervention' OR 'coronary artery bypass graft'/exp OR 'coronary artery bypass graft' OR 'heart failure'/exp OR 'heart failure' OR 'cardiovascular disease*':ti,ab OR 'coronary heart disease*':ti,ab OR 'coronary artery disease*':ti,ab OR 'acute coronary syndrome*':ti,ab OR 'myocardial infarction*':ti,ab OR 'percutaneous coronary intervention*':ti,ab OR 'pci':ti,ab OR 'coronary artery bypass*':ti,ab OR 'cabg':ti,ab OR 'heart failure':ti,ab OR 'cardiac failure':ti,ab) AND ('heart rehabilitation'/exp OR 'heart rehabilitation' OR 'cardiovascular rehabilitation'/exp OR 'cardiovascular rehabilitation' OR 'telerehabilitation'/exp OR 'telerehabilitation' OR 'home-based rehabilitation' OR 'cardiac rehabilitation':ti,ab OR 'heart rehabilitation':ti,ab OR 'cardiovascular rehabilitation':ti,ab OR 'cardiac rehab':ti,ab OR 'heart rehab':ti,ab OR 'exercise-based rehabilitation':ti,ab OR 'telerehabilitation':ti,ab OR 'home-rehabilitation':ti,ab) AND [clinical study]/lim AND [adult]/lim AND [randomized controlled trial]/lim AND 'article'/it AND [humans]/lim AND [embase]/lim

**Cochrane Search Strategy**

([mh "Telephone"] OR "telephone":ti,ab OR [mh "Cell Phone"] OR "cell phone":ti,ab OR [mh "Smartphone"] OR "smartphone":ti,ab OR [mh "Text Messaging"] OR "text messaging":ti,ab OR "message":ti,ab OR [mh "Internet"] OR "internet":ti,ab OR [mh "Online Social Networking"] OR "online social networking":ti,ab OR [mh "Mobile Applications"] OR "mobile applications":ti,ab OR [mh "Remote Consultation"] OR "remote consultation":ti,ab OR [mh "Distance Counseling"] OR "distance counseling":ti,ab OR [mh "Telemedicine"] OR "telemedicine":ti,ab ) AND ([mh "Cardiovascular Diseases"] OR [mh "Heart Diseases"] OR [mh "Coronary Artery Disease"] OR [mh "Myocardial Infarction"] OR [mh "Heart Failure"] OR [mh "Percutaneous Coronary Intervention"] OR [mh "Coronary Artery Bypass"]) AND ([mh "Cardiac Rehabilitation"] OR "cardiac rehabilitation":ti,ab OR "heart rehabilitation":ti,ab OR "cardiovascular rehabilitation":ti,ab OR "cardiac rehab":ti,ab OR "heart rehab":ti,ab OR "exercise-based rehabilitation":ti,ab OR "telerehabilitation":ti,ab OR "home-based rehabilitation":ti,ab OR "home rehabilitation":ti,ab OR "remote rehabilitation":ti,ab)

**Supplementary Section 2:** Forest plots of the effects of RCR on health-related quality of life (HRQoL) in cardiovascular patients.

Figure S1. Forest plots of the effects of RCR on Hospital Anxiety and Depression Scale (HADS) domains.

(A) Pooled analysis of the HADS anxiety subscales at the 6-month and 12-month follow-up.

(B) Pooled analysis of the HADS depression subscales at the 6-month and 12-month follow-up.

**Supplementary Section 3:** Forest plots of the effects of RCR on cardiovascular risk profiles and follow-up durations.

Figure S2. Forest plot detailing the subgroup analysis of the risk of major adverse cardiovascular events (MACEs), subgroup analysis by short-term, medium-term, and long-term follow-up durations.

Figure S3. Forest plots detailing the risk of safety outcomes associated with RCR. (A) Pooled analysis of the risk of all-cause mortality; (B) Pooled analysis of the risk of all-cause adverse events.

Figure S4. Forest plot detailing the subgroup analysis of the risk of cardiac rehospitalizations, stratified by cardiovascular risk profile.

Figure S5. Forest plot detailing the subgroup analysis of the risk of cardiac rehospitalizations, stratified by short-term, medium-term, and long-term follow-up durations.

Figure S6. Forest plot detailing the subgroup analysis of the risk of cardiac rehospitalization, stratified by intervention modalities.

Figure S7. Sensitivity analysis plots across all clinical outcomes.

(A) SF-36 overall score; (B) SF-36 physical health score; (C) SF-36 mental health score; (D) MacNew physical score; (E) MacNew emotional score; (F) MacNew global score; (G) HADS anxiety score; (H) HADS depression score; (I) Minnesota Living with Heart Failure Questionnaire score; (J) MACEs; (K) Cardiac rehospitalization; (L) All-cause mortality; (M) All-cause adverse events.

Figure S8. Funnel plots for the publication bias across all clinical outcomes.

(A) SF-36 overall score; (B) SF-36 physical health score; (C) SF-36 mental health score; (D) MacNew physical score; (E) MacNew emotional score; (F) MacNew global score; (G) HADS anxiety score; (H) HADS depression score; (I) Minnesota Living with Heart Failure Questionnaire score; (J) Major adverse cardiovascular events (MACEs); (K) Cardiac rehospitalization; (L) All-cause mortality; (M) All-cause adverse events.

**Supplementary Section 4:**

Trial sequential analyses (TSA) for health-related quality of life and cardiovascular adverse events.

Figure S9. TSA evaluating the robustness of HRQoL outcomes. (A) TSA for the SF-36 overall score; (B) TSA for the MacNew global score.

The cumulative Z-curve for the SF-36 overall score fails to reach the estimated RIS (n=1974), suggesting that although preliminary trends are observable, the accrued sample size remains insufficient to firmly establish a definitive conclusion (Figure S8A). The cumulative Z-curve for the MacNew global score conclusively crosses both the conventional boundary and the trial sequential monitoring boundary, whilst also surpassing the estimated RIS threshold (n=354). This strongly indicates that the current cumulative evidence is robust and statistically conclusive.
