# Supplementary material for: Long-term quality of life, rehospitalization risks, and economic viability of remote cardiac rehabilitation: an updated systematic review and meta-analysis
Source: Front Digit Health. 2026 Oct 14;8:1935832. doi: 10.3389/fdgth.2026.1935832 (PMC13713463; doi:10.3389/fdgth.2026.1935832)
Supplement: Supplementary file 2 [file Datasheet2.docx]

**Supplementary Table S1. Study characteristics of the included studies**

| **Study & Country** | **Study Type Trial Reg. Number** | **Study Design/Intervention Details** | | **Study Population Follow-up** | **Follow-up** | **Key Outcomes** | **Safety/Events** | **Medical Costs** |
| --- | --- | --- | --- | --- | --- | --- | --- | --- |
| Bae JW et al. (2021) Korea | Parallel, single-blind, 1:1 RCT  KCT0005087 | Standard care + SMS & Web: Usual care plus web access and 4 SMS/week (96 total) for 6 months, covering diet, activity, smoking, and meds. | Standard care: Guideline-directed care (medication, outpatient follow-up, nurse education). | N=879 (age 60.4, 83% male). CHD, post-1st PCI. | 6 months | Behavioral: Significant improvement in physical activity (P=.02), fruit/vegetable intake (P=.006), and medication adherence (P<.001) . Physiological/Clinical: No significant differences between groups in LDL-C (P=.12), systolic blood pressure (P=.13), or BMI (P=.41) . | Mortality: 4 in intervention vs 1 in control. | Not reported |
|  |  |  |  |  |  |  |  |  |
| Chow CK et al. (2022) Australia | Single-blind, multicenter RCT  ACTRN12613000793718 | TEXTMEDS: 12-month text-based program (4x/week for months 1-6; 3x/week for months 7-12) for customized secondary prevention & 2-way communication. | Usual care: Secondary prevention (welcome message and follow-up alerts only). | N=1424 (age 58, 79% male). Post-ACS. | 6 months and 12 months | Primary outcome: No significant difference in self-reported medication adherence (≥80% adherence to up to 5 indicated medications) at 6 and 12 months between groups (RR 0.93, p=0.15). No difference in individual medication adherence, BP, LDL-C, smoking, or regular exercise. | No significant difference in clinical events. | Not reported |
|  |  |  |  |  |  |  |  |  |
| Claes J et al. (2020) Belgium & Ireland | Single-blind, multicenter pilot RCT  NCT02717806 | PATHway: Home-based, remotely monitored CR with telemonitoring, e-learning, telecoaching, and Exergames/social networking after 4-week familiarization. | Usual care: Verbal advice on physical activity and lifestyle. | N=120 (age 61.4, 82% male). CVD, post-Phase II center-based CR. | 6 months | Moderate-to-vigorous physical activity (MVPA) increased significantly in the PATHway group (127 to 141 min) but decreased slightly in the usual care group, resulting in a significant interaction effect (p=0.04). Diastolic BP and cardiovascular risk scores deteriorated in usual care but remained stable in the intervention group. No significant differences in physical fitness (VO2 peak), quality of life, or other risk factors between groups. | Not explicitly evaluated. | Not reported |
|  |  |  |  |  |  |  |  |  |
| De Lima AP et al. (2022) Brazil | Single-blind RCT  NCT03605992 | Home CR: 12-week program (6 education + 2 supervised center sessions + 58 home sessions) monitored via HR/pedometer and weekly phone calls. | Traditional CR: 12-week traditional center-based CR. 24 center-based supervised training sessions + 36 home-based unsupervised sessions, with the same educational program. | N=49 (age ~56.4, ~85% male). CAD (post-MI/PCI/CABG), low-moderate exercise risk. | 12 weeks (post-CR) and 24 weeks (12 weeks after completion) | Behavioral/Physiological: No significant difference in adherence between Home-CR (94%) and TCR (79%). Functional capacity improved post-CR and was maintained at follow-up in both groups, with no difference between groups. Risk factors and quality of life showed no significant differences between groups. | No significant difference in morbidity or hospitalizations. Lower cost in the intervention group. | Cost per patient was lower in the Home-CR group (US$ 59.31 vs US$ 135.05). |
|  |  |  |  |  |  |  |  |  |
| Devi R et al. (2014) United Kingdom | RCT  ISRCTN90110503 | Web-based CR (ActivateYourHeart): 6-week online program (tailored goals, exercise diary, specialist communication) initiated face-to-face. | Treatment as usual: GP care (CHD register and annual review). | N=94 (age ~66.2, ~74% male). Stable angina (from primary care). | 6 weeks and 6 months (after 6-week follow-up) | Behavioral/Physiological: At 6 weeks, the intervention group showed significant improvements in daily steps walked (primary outcome), energy expenditure, duration of sedentary/moderate activity, weight, self-efficacy, emotional QOL, and angina frequency. Control group had a greater reduction in systolic BP. At 6 months, significant benefits in angina frequency and social QOL remained in the intervention group, but physical activity differences were no longer significant. | Not explicitly evaluated. | Not reported |
|  |  |  |  |  |  |  |  |  |
| Gallagher R et al. (2022) Australia | Parallel-group, two-arm, single-blind RCT  ACTRN12617000869370 | Game-based App (MyHeartMate): 6-month app usage (heart avatar, tokens earned via tracking health metrics, quizzes, and challenges). | Usual care: Follow-up visits and CR referral. | N=390 (age ~61.2, ~83% male). CHD (MI/angina/CABG/PCI or >50% stenosis). | 6 months | Behavioral/Physiological: No statistically significant difference in the primary outcome of physical activity (MET mins/wk) (p=0.064). No differences in most secondary outcomes (BMI, BP, LDL-C, meeting guidelines) except for significantly lower triglyceride levels in the intervention group. | Not explicitly evaluated. | Not reported |
|  |  |  |  |  |  |  |  |  |
| Hawkes AL et al. (2013) Australia | Prospective, parallel-group RCT  ACTRN12607000595415 | Telephone CR (ProActive Heart): Up to 10 scripted, 30-min telephone coaching sessions over 6 months based on social cognitive theory. | Usual care: Educational resource and quarterly newsletter. | N=430 (age ~60.6, ~75% male). Post-MI. | 6 months | Behavioral/Physiological: Intervention group showed significantly greater improvements in health-related quality of life (SF-36 mental component summary score, social functioning, and role-emotional subscales). Intervention participants were also significantly more likely to meet recommended guidelines for physical activity, BMI, vegetable intake, and alcohol intake. | Not explicitly evaluated. | Not reported |
|  |  |  |  |  |  |  |  |  |
| Kamel H et al. (2021) Egypt | RCT  Not specified | Smartphone Video Consultation: 3-month monthly free-of-charge video consultations (Zoom) to review lifestyle/meds + ≥1 face-to-face visit. | Usual care: ≥1 face-to-face clinic visit within 3 months. | N=200 (age ~56, ~71% male). STEMI, post-primary PCI. | 4 months (after discharge) | Behavioral/Physiological: Intervention group had significantly better adherence to statins, ACEI/ARB, smoking cessation, and cardiac rehabilitation. High patient satisfaction (87%) with telemedicine. | No significant difference in short-term major adverse cardiovascular events (11% intervention vs 14% control). | Not reported |
|  |  |  |  |  |  |  |  |  |
| Kraal JJ et al. (2017) Netherlands | RCT  NCT01732419 | Home-based CR with Telemonitoring: 3-month home training (2x/week) via HR monitor and web app, plus weekly motivational phone feedback, after 3 intro center sessions. | Conventional CR: 3-month center-based supervised training (≥2x/week). | N=90 (age ~59, 89% male). Post-ACS/revascularization entering CR, low-moderate risk. | Discharge (approx. 3 months) and 1 year (12 months) | Behavioral/Physiological: Both groups significantly improved physical fitness (peak VO2) at discharge and maintained it at 1 year, with no significant differences between groups. Physical activity levels did not change significantly in either group. HRQoL and psychological status were similar between groups. Patient satisfaction was significantly higher in the home-based group. | 2 rehospitalizations in the intervention group vs 8 in the control group. | Telemonitored home-based CR proved more cost-effective than center-based CR. While yielding similar QALYs (p=0.73), it showed a trend toward lower total healthcare costs (-€437, p=0.392) and societal costs (-€3160, p=0.087), primarily driven by significantly reduced physician visits (p<0.05) and work absenteeism. |
|  |  |  |  |  |  |  |  |  |
| Lao SSW et al. (2023) Macao, China | Single-blind RCT  ChiCTR1800014521 | Mobile Health CR (mCR): App-based education, self-monitoring, chatroom, and phone calls at weeks 4 and 9 based on social cognitive theory. | Usual care: CR booklet and written logbooks. | N=140 (median age 68-70, ~78% male). CHD, post-PCI. | 6 weeks and 12 weeks | Behavioral/Physiological: The intervention group showed significant improvements in total cholesterol, LDL, 6-minute walk test, sitting time reduction, regular exercise performance, self-efficacy (cardiac exercise and diet), anxiety level, total HADS score, and quality of life at 12 weeks. No significant effect on BMI, weight, BP, smoking, or glycemic control. | No significant difference in 30-day and 90-day cardiac readmissions. | Not reported |
|  |  |  |  |  |  |  |  |  |
| Leemrijse CJ et al. (2016) Netherlands | Multicenter, parallel-group RCT  NTR2388 | Hartcoach: Usual care + telephonic lifestyle coaching (every 4-6 weeks for 6 months) to stimulate self-management. | Usual care: Standard multi-disciplinary care visits. | N=374 (age 60.4, 81% male)，<8 weeks post-AMI or angina. | 6 months | Behavioral/Physiological: Intervention group had significant favourable effects on BMI, waist circumference, physical activity (PASE score), daily intake of vegetables, self-management, and anxiety compared to control. No significant effects on cholesterol, blood pressure, smoking, or glucose. | Not explicitly evaluated. | Not reported |
|  |  |  |  |  |  |  |  |  |
| Li X et al. (2023) China | Single-blind pilot RCT  ChiCTR2300074435 | Cardiac Telerehabilitation (CTR): 3-month, 3x/week program using 5G IoT platform, wearables, and traditional Chinese exercise (Ba Duan Jin). | Center-based CR: In-hospital CR training + Ba Duan Jin. | N=50 (age 66.3, 48% male)， AMI, post-PCI. | 3 months (end of intervention) | Behavioral/Physiological: Intervention group showed significantly greater improvements in VO2max, MET, total cholesterol, triglycerides, HDL-C, BMI, and psychological indicators (GAD-7, PHQ-9) compared to control. No significant difference in LDL-C. Compliance and satisfaction were significantly higher in the intervention group. | No significant difference in major adverse cardiovascular events. | Not reported |
|  |  |  |  |  |  |  |  |  |
| Ögmundsdóttir Michelsen H et al. (2022) Sweden | Multicenter RCT  NCT03260582 | Center CR + Web App ('LifePod'): 25-week program combining center CR with an app for logging health metrics and receiving automated feedback. | Usual care: Comprehensive center-based CR (5 outpatient visits). | N=150 (age ~60.5, 77% male)，<2 weeks post-MI. | 2 weeks, 6-10 weeks, 4-6 months, and 12-14 months | Behavioral/Physiological: Intervention group had a significantly larger reduction in systolic BP at 2 weeks and 6-10 weeks, and a significantly larger reduction in diastolic BP at 6-10 weeks. Healthy diet index improved significantly more in the intervention group at 2 weeks. No significant difference in submaximal exercise capacity. | Similar rehospitalizations (All-cause: 22% intervention vs 27% control; Ischemic: 8% intervention vs 4% control). | Not reported |
|  |  |  |  |  |  |  |  |  |
| Pakrad F et al. (2021) Iran | RCT  IRCT20130211012439N3 | Extended Hybrid CR: 1-month supervised in-person CR + 3-month remote support via app (biweekly nurse communication, education, targets). | Traditional CR: 1-month supervised group CR (3x/week). | N=88 (age 62.8, 84% male). Post-CABG. | 1 month (post-supervised CR) and 4 months (3 months after supervised CR end) | Behavioral/Physiological: Intervention group had significant improvements in QOL (Physical and Mental Component Summary scores), functional capacity (1.5 MET increase), and psychosocial wellbeing (depression, anxiety, stress) compared to the control group at 4 months. | Rehospitalizations were significantly lower in the intervention group (0% vs 10.3%, P=.049). | Not reported |
|  |  |  |  |  |  |  |  |  |
| Patterson K et al. (2023) Australia | Multicenter RCT  ACTRN12619001223123 | ToDo-CR: Usual care CR + 6-month app-based (Vire) program using ML/wearables to send personalized notifications to break sedentary habits. | Usual care CR: 5-6 weeks of standard face-to-face, hybrid, or telehealth sessions. | N=120 (age 62.6, 78% male). Stable CHD in Phase II CR. | 6 and 12 months | Behavioral/Physiological: No significant between-group difference in device-measured sedentary behavior at 12 months. Significant improvements in BMI, waist circumference, and quality of life in the intervention group compared to control. | No significant difference in all-cause/cardiac admissions and emergency department visits. | The intervention (Aus $1086.55/patient) was not cost-effective; despite non-significant savings in 12-month cardiac admissions, it significantly increased 6-month ED costs (P=.04) and yielded high ICERs for clinical improvements. |
|  |  |  |  |  |  |  |  |  |
| Pfaeffli Dale L et al. (2015) New Zealand | RCT  ACTRN 12613000901707 | Text4Heart: 24-week program of daily automated SMS and website support (based on social cognitive theory) + usual care. | Usual care: Inpatient CR + encouragement for 6-week outpatient CR. | N=123 (age 59.5, 81% male). CHD. | 3 and 6 months | Behavioral/Physiological: Intervention group showed a significantly greater adherence to recommended lifestyle behavior changes (composite score ≥3) at 3 months (AOR 2.55, P=.03), but this was not sustained at 6 months (P=.13). Medication adherence was significantly higher in the intervention group at 6 months. | 13 serious adverse events (none study-related). No significant difference in clinical outcomes. | Not reported |
|  |  |  |  |  |  |  |  |  |
| Pinto BM et al. (2013) USA | RCT  Not specified | Home-based exercise maintenance counseling via phone + print materials, reports, and pedometer. | Contact Control: Phone calls to monitor general health + tip-sheets. | N=130 (age 63.6, 79% male). Post-Phase II CR. | 6 and 12 months | Behavioral/Physiological: MC group reported significantly higher quality of life (MacNew) at 6 and 12 months, and significantly reduced depressive symptoms (Cardiac Depression Scale) at 12 months compared to CC. No significant effects on general mental health (SF-36) at either follow-up. | 1 death reported in each group. | Not reported |
|  |  |  |  |  |  |  |  |  |
| Seron P et al. (2024) Chile | Pragmatic, multicenter, parallel-arm, open-label RCT  NCT03881150 | Hybrid CR: 10 supervised center sessions + counseling (weeks 1-6) followed by home support via phone/SMS (weeks 8-12). | Standard CR: 18-22 supervised center sessions over 8-12 weeks. | N=191 (age 58.7, 76% male). CAD (post-event/procedure). | 6 and 12 months (up to 30 months maximum) | Behavioral/Physiological: No significant between-group differences for secondary outcomes (QoL, functional capacity, muscle strength) except for a significantly higher adherence to supervised sessions in the Hybrid CR group (79.1% vs 61.5%). analysis. | Cardiovascular events and mortality were non-inferior in the intervention group (5.4%) vs the control group (9.2%). | Not reported |
|  |  |  |  |  |  |  |  |  |
| Skobel E et al. (2017) Germany/Spain/UK | Prospective, multicenter RCT  NCT01761448 | Smartphone-guided CR: Home-based training via a wearable sensor (ECG/breathing) for immediate feedback, transmitting data to a medical team. | Standard Home CR: National standard using paper diaries for activity. | N=118 (age 58, 89% male). CAD post-MI/intervention (LVEF ≥30%). | 6 months | Behavioral/Physiological: IG demonstrated a significantly higher improvement in peak oxygen uptake (VO2peak) compared to CG (1.76 vs -0.4 ml/min/kg, P=0.005). Significant improvements in EF and resting blood pressure parameters favored IG. | Safe (no complications related to cardiac rehabilitation). | Not reported |
|  |  |  |  |  |  |  |  |  |
| Snoek JA et al. (2021) Europe (5 countries) | Parallel, multicenter RCT  NL5168 | Mobile CR (MCR): 6-month home-based program (≥30min/day, 5 days/week) via telemonitoring (app & HR belt) + motivational telephone coaching. | Locally defined standard of care without any form of cardiac rehabilitation. | N=179 (median age 72, age ≥65). CAD/valvular intervention, declining center-based CR. | 6 and 12 months | Behavioral/Physiological: MCR group showed significantly greater improvement in peak oxygen uptake (VO2 peak) at 6 and 12 months compared to control. Self-reported physical activity also increased more in the MCR group. | No significant difference in serious adverse events (13% intervention vs 11% control). | Not reported |
|  |  |  |  |  |  |  |  |  |
| Sunamura M et al. (2018) Netherlands | Open-label superiority RCT  NCT01395095 | CR + Face-to-Face (CRF) / CR + Telephone (CRT): 3-month standard CR + either 3 extended face-to-face group sessions OR 5-6 individual phone sessions. | CR-only: 3-month standard CR (group exercise 2x/week + education). | N=914 (age 57, 81% male). ACS. | 18 months | Primary Outcome: No significant difference in SCORE risk function at 18 months between CRF (3.30) and CR-only (3.47) (P=0.48), or CRT (3.02) and CR-only (3.47) (P=0.39). Secondary/Per-protocol: CRF group showed lower current smoking rates (13.4% vs 21.3%, P<0.05) and total cholesterol vs CR-only. CRT also had a lower smoking rate (12.9% vs 21.3%, P<0.05). | No significant difference in rehospitalizations. | Not reported |
|  |  |  |  |  |  |  |  |  |
| Widmer RJ et al. (2017) USA | Prospective RCT  NCT01883050 | CR + Digital Health Intervention (DHI): 3-month standard CR + online/app platform for logging habits and receiving education. | Standard CR: 3-month standard CR alone. | N=80 (age ~63). Post-ACS/PCI undergoing CR. | 90 days for health metrics, 180 days for MACE/ED visits. | Behavioral/Physiological: DHI+CR group had significantly improved weight loss (-5.1 kg vs. -0.8 kg, P=0.02), BMI reduction, waist circumference reduction, and improved diet score at 90 days. | Non-significant reduction in cardiovascular rehospitalizations and emergency department visits (8.1% intervention vs 26.6% control, P=.054). | Not reported |
|  |  |  |  |  |  |  |  |  |
| Yudi MB et al. (2021) Australia | Single-blinded, two-arm, parallel, RCT  ACTRN12616000426482 | Smartphone CR (S-CRP): App as an adjunct to usual care upon discharge, tracking risk factors dynamically with interactive feedback. | Usual care: Traditional care including referral to CR. | N=206 (age 56, 84% male). ACS (168 completed follow-up). | 8 weeks | Behavioral/Physiological: S-CRP group had significantly improved exercise capacity measured by a 6-minute walk test distance at 8 weeks (Δ117m vs. Δ91m, P=0.02). S-CRP group was more likely to participate (87% vs. 51%, P<0.001) and adhere (72% vs. 22%, P<0.001) to cardiac rehabilitation. | No significant difference in major adverse cardiovascular events. | Not reported |
|  |  |  |  |  |  |  |  |  |
| Li Z et al. (2022) China | Parallel RCT  Not specified | Remote Home CR: Home walking program with real-time wearable/app monitoring, daily WeChat supervision, and online follow-up. | Traditional CR: Outpatient center-based rehabilitation. | N=80 (age ~55, ~66% male). AMI, post-emergency PCI. | 6 months | Behavioral/Physiological: At 6 months, the intervention group had significantly better walking compliance (95% vs. 50%, P<0.05), medication compliance, 6-minute walking test distance (421.7m vs. 346.7m, P<0.05), and greater LDL reduction (P<0.05) compared to control. | Significantly lower heart failure, unstable angina, and unplanned readmissions in the intervention group. No significant difference in fatality or second myocardial infarction. | Not reported |
|  |  |  |  |  |  |  |  |  |
| Nagatomi Y et al. (2022) Japan | Single-centre, open-label, RCT  UMIN000040136 | ICT Home-based CR (HBCR): 3-month program with exercise guidance (wearable/app) and individual nutritional guidance via meal photos. | Standard Care: Standard medical therapy without HBCR. | N=30 (age ~63.7, 53% male). Chronic HF (NYHA II-III) with physical frailty. | 3 months | Behavioral/Physiological: HBCR group showed a significantly greater increase in 6-minute walking distance (6MWD) compared to control (+52.1m vs. -4.3m, P<0.001). Significant improvements were also seen in knee extension strength and serum total cholesterol in the HBCR group. | No significant difference in adverse events. 3 readmissions per group. | Not reported |
|  |  |  |  |  |  |  |  |  |
| Olivier CB et al. (2021) USA/Germany | Open-label, single-center RCT  NCT03207646 | smartGUIDE: BrightHeart app providing virtual health coaching combined with home-based CR components. | Usual care: Physician- or nurse-guided care. | N=63 (26 analyzed). CAD prescribed statin/P2Y12 inhibitor (trial terminated early). | 90 ± 10 days | Behavioral/Physiological: No significant difference in medication adherence (PDC ≥ 80% was 82% in intervention vs. 93% in control, P=0.56). Median PDC was 94% in intervention vs. 99% in control (P=0.034). No significant differences in body weight, self-reported physical activity, or quality of life. | Trial terminated early (technology startup folded). | Not reported |
|  |  |  |  |  |  |  |  |  |
| Su JJ et al. (2021) China | Single-blinded RCT  ChiCTR1800020411 | Nurse-led eHealth CR (NeCR): 12-week program (in-person goal setting + post-discharge website/WeChat for tracking and weekly nurse/peer feedback). | Usual care: 10-min didactic session at discharge + pedometer. | N=146 (age ~55.8, 84% male). Hospitalized for CHD (73% post-PCI). | 6 and 12 weeks | Behavioral/Physiological: NeCR significantly improved daily steps, weekly sitting minutes, health-promoting lifestyle profile, smoking cessation, self-efficacy, and physical/social domains of quality of life (MacNew) at 12 weeks compared to control. No significant effect on BMI, blood pressure, or psychological wellbeing (DASS-21). | No significant difference in unplanned admissions (2 per group). | Not reported |
|  |  |  |  |  |  |  |  |  |
| Bernocchi P et al. (2018) Italy | Open, multicenter RCT  NCT02269618 | Home telerehabilitation (weekly nurse/physiotherapist calls, ECG/SpO2 telemonitoring, tailored exercise) . | Control: Standard care. | 112 older patients (mean age 70) with combined COPD and CHF post-hospitalization. | 6 months | Significant improvement in 6MWT (+60m vs -15m, P=0.0040) , dyspnea, physical activity, disability, and QoL in intervention group . | Longer time to hospitalization/death in intervention group (113.4 vs 104.7 days, P=0.0484). | Not reported |
|  |  |  |  |  |  |  |  |  |
| Frederix I et al. (2016) Belgium | Multicenter RCT  ISRCTN29243064 | 24-week internet-based telerehabilitation (wearable accelerometer telemonitoring, email/SMS coaching for diet/smoking/activity) plus 12-week standard center-based CR. | Control: 12-week standard center-based CR alone. | 140 patients entering CR for coronary artery disease (CAD) or chronic heart failure (CHF). | 12 months | Significantly improved quality-adjusted life years (QALYs: +0.06 vs -0.09) . | Significantly prolonged time to first CV rehospitalization (502 vs 445 days, P=0.045) and fewer days lost to CV rehospitalization (0.33 vs 0.79 days, P=0.037). | Significantly lower in the intervention group total cost (€2156 vs €2720, P=0.01); highly cost-effective (ICER: €-21,707/QALY). |
|  |  |  |  |  |  |  |  |  |
| Hwang R et al. (2019) Australia | Randomised, parallel, non-inferiority trial  ACTRN12613000390785 | 12-week home-based telerehabilitation via real-time online videoconferencing in small groups (twice weekly, 60-min exercise and education sessions) . | Control: 12-week traditional hospital outpatient center-based rehabilitation (twice weekly, 60-min sessions). | 53 patients with stable chronic heart failure (reduced or preserved ejection fraction). | 6 months | Non-inferior to control for 6MWD at 12 weeks. Similar functional capacity, strength, and QoL. Significantly higher program attendance .No significant difference in quality-adjusted life years (QALYs) between groups (MD=0; 95% CI: -0.06 to 0.05). | No significant difference in adverse events between groups. No deaths, cardiac arrests, syncopes, or falls occurred during exercise sessions. | Total health care cost per participant was significantly lower in the telerehabilitation group (AUD 2,325 vs 3,916; difference: -$1,590). Highly cost-effective with an ICER of -$4,157/QALY. |
|  |  |  |  |  |  |  |  |  |
| Indraratna P et al. (2022) Australia | Pilot, 2-center RCT  ACTRN12618001547235 | TeleClinical Care (TCC) smartphone app + Bluetooth devices (BP, weight, activity). Included daily telemonitoring with automated clinical alerts, plus educational push notifications. | Usual care alone. | 164 patients discharged after admission for acute coronary syndrome (ACS) or heart failure (HF). | 6 months | Significantly higher rates of CR completion (39% vs 18%, P=0.03) and medication adherence (75% vs 50%, P=0.002). HRQoL (EQ-5D) improved in both groups with no significant between-group difference. | Significantly fewer 6-month unplanned all-cause readmissions (21 vs 41, P=0.02) and cardiac readmissions (11 vs 25, P=0.03). No significant difference in 30-day readmissions or mortality. | Although reducing cardiac readmission costs, the total intervention cost (Aus $82,408) was higher, resulting in a net cost of Aus $6,028 per cardiac readmission avoided during the 20-month trial. |
|  |  |  |  |  |  |  |  |  |
| Kitsiou S et al. (2025) USA | Phase 1 RCT (Feasibility/Pilot)  NCT03642275 | 8-week patient-centered mHealth intervention (iCardia4HF) utilizing 3 apps and 3 wearable/smart devices for daily telemonitoring (BP, weight, activity), plus tailored SMS text messages. | Usual care alone. | 25 analyzed patients with stage C chronic heart failure (NYHA class I-III). | 2 months | Improved HF self-care scores (SCHFI) and health status (KCCQ-12) at 8 weeks. High adherence to daily self-monitoring (>86%). | Zero deaths or intervention-related adverse events, with low hospitalizations (all-cause: 4 vs 2; HF: 1 vs 0). | Not reported. |
|  |  |  |  |  |  |  |  |  |
| Lundgren KM et al. (2025) Norway | RCT  NCT03183323 | 3-month home-based high-intensity telerehabilitation via real-time videoconferencing (twice weekly) + educational HF course. | Educational HF course only. | 61 chronic heart failure (CHF) patients who declined or were unable to attend standard outpatient CR. | 24 months | No significant long-term difference in physical activity (MVPA) or functional capacity ($VO_{2peak}$) between groups. Both groups showed significant within-group improvements in HRQoL (MLHFQ). | No significant difference in time to HF hospitalization or death (P=0.54). Low mortality (1 death in intervention vs 4 in control). | Not reported. |
|  |  |  |  |  |  |  |  |  |
| Nabutovsky I et al. (2024) Israel | RCT  Not reported | 6-month asynchronous home-based CR via smartwatch and smartphone app, featuring structured exercise, remote telemonitoring, education, and multidisciplinary team follow-up. | Usual care (general advice, periodic consults, and app for step counting only). | 69 moderate-to-high risk patients (post-MI, PCI, CABG, HF, or transplant) who actively declined/were unwilling to participate in center-based CR. | 6 months (Primary outcome at 4 months) | HBCR significantly improved VO2peak (+10.2% vs -2.7%, P<.001) and HDL compared to control at 4 months. High exercise adherence. No significant difference in HRQoL . | Safe intervention with zero deaths and equal, low rates of non-intervention-related short hospitalizations (2 in HBCR vs 2 in control). | Not reported. |
|  |  |  |  |  |  |  |  |  |
| Peng X et al. (2018) China | RCT  Not reported | Home-based telehealth exercise training (32 sessions of aerobic and resistance exercises) via WeChat/QQ for real-time supervision and regular follow-ups . | Usual care alone. | 98 clinically stable heart failure patients (NYHA class I-III). | 2 months | Telehealth CR significantly improved QoL (MLHFQ, P=0.005), functional capacity (6MWD, P<0.001), and resting HR, but did not alter LVEF, NYHA, or psychological status. | No significant complications/adverse outcomes. | Not reported. |
|  |  |  |  |  |  |  |  |  |
| Piotrowicz E, Irzmański R, Mierzyńska A et al. (2020-2023) Poland | Multicenter RCT (TELEREH-HF)  NCT02523560 | Hybrid comprehensive telerehabilitation (HCTR). | Usual care alone. | Female patients with heart failure (a predefined subgroup derived from the main TELEREH-HF trial). | 9 weeks (Functional); 12-24 months (Median 22 months for clinical events). | HCTR significantly improved exercise capacity (Peak VO2 and 6MWT; both P<0.001) and physical QoL domains (SF-36), yielding incremental QALY gains of 0.044 (NYHA-based) and 0.027 (SF-36-based). These benefits were consistent in women (improved Peak VO2, workload, and QoL). However, HCTR did not improve echocardiographic diastolic function. | No significant differences in CV mortality (9.9% in HCTR vs. 9.7% in UC, P=0.91) or HF hospitalizations (22.8% vs. 20.2%, P=0.36). | Incremental cost of telerehabilitation was 2,581 PLN. The ICUR was 58,663 PLN/QALY (NYHA) and 95,955 PLN/QALY (SF-36). Both are well below the national threshold, proving HCTR is highly cost-effective. |
|  |  |  |  |  |  |  |  |  |
| Piotrowicz E et al. (2010/2015) Poland | RCT  Not reported | Home-based telehealth exercise training (32 sessions of aerobic and resistance exercises) via WeChat/QQ for real-time supervision and regular follow-ups . | Standard outpatient CR (SCR) with cycloergometer. | 152 patients with clinically stable heart failure (NYHA II-III, LVEF ≤ 40%). | 2 months | Both groups equally improved Peak VO_2 (P=0.0001) and total QoL. Interestingly, HTCR yielded superior NYHA (P=0.007), adherence (100% vs 80%), and mental QoL (MCS), whereas SCR showed superior 6MWD (P=0.047) and physical QoL (PCS). | No significant complications/adverse outcomes. | Not reported. |
|  |  |  |  |  |  |  |  |  |
| Piotrowicz E et al. (2015b) Poland | RCT (2:1 ratio)  Not reported | Home-based telemonitored Nordic walking (NW) training (5 sessions/week). | Usual care alone. | 111 stable HF patients (NYHA II-III, LVEF≤ 40%), including many with CIEDs (ICDs/CRT). | 2 months | NW significantly improved Peak V2 (P=0.0004), 6-MWT distance (P=0.0483), workload (P=0.0031), and QoL (SF-36). | Highly safe (0 deaths, hospitalizations, AEs, or CIED interventions) with 94.7% adherence. | Not reported. |
|  |  |  |  |  |  |  |  |  |
| Schmidt C et al. (2025) Portugal | RCT (Noninferiority)  NCT04334603 | 12-week home-based CR (4 supervised center sessions + 20 home sessions asynchronously telemonitored via smartwatch and weekly phone calls). | 12-week standard center-based CR (24 supervised sessions). | 120 HF patients (mostly HFrEF, mean LVEF =36±11%). | 3 months | Demonstrating noninferiority to center-based CR, home-based CR resulted in equally significant improvements in VOpeak (P=0.10), 6MWT (P=0.73), and HR-QoL (P=0.97). | No significant complications/adverse outcomes. | Not reported. |
|  |  |  |  |  |  |  |  |  |
| Smolis-Bąk E et al. (2015) Poland | RCT  Not reported | Home-based CR (5 sessions/week) telemonitored via mobile phone (ECG, weight, and BP transmission). | Standard hospital-based CR (3 sessions/week). | 100 patients with advanced HF (NYHA class III, LVEF≤35%) and CRT-D. | 2 months | Both groups showed equal and significant improvements in Peak VO2 (P<0.01), 6MWT distance (P<0.01), and MLHFQ scores (P<0.01). | Zero exercise-related deaths or CRT-D device-related adverse events. | Not reported. |
|  |  |  |  |  |  |  |  |  |
| Vetrovsky T et al. (2024) Czech Republic | Multicenter RCT  (WATCHFUL Trial)  NCT03041610 | Lifestyle walking intervention combining self-monitoring (using a pedometer/wearable) and regular telephone counseling. | Usual care alone. | 202 patients with stable HFrEF (LVEF < 40%, NYHA II-III). | 6 months | Significantly increased daily steps (+1420) but yielded neutral 6MWT results (P=0.345) with no functional improvement. | No intervention-related adverse events. HF hospitalizations: 1 (intervention) vs 3 (control). | Not reported. |
|  |  |  |  |  |  |  |  |  |
| Taylor JL et al. (2020) Australia | RCT (FITR Heart substudy)  ACTRN12615001292561 | HIIT: 4-week supervised center-based CR (3 sessions/week) followed by an 11-month home-based maintenance program. Protocol: 4 times 4-min HIIT at RPE 15-18 (85%-95% HRpeak), interspersed with 3-min active recovery | MICT (Usual care): Same frequency and duration. Protocol: 34-min MICT at RPE 11-13 (65%-75% HRpeak). | N=42 (mean age ~64 years, ~81% male). Patients with coronary artery disease (CAD) following a recent acute cardiac event or revascularization. | 3 months and 12 months | Both interventions significantly reduced visceral and liver fat over 12 months with no differences in weight or BMI, though higher exercise intensity independently predicted greater liver fat reduction. | Three serious adverse events requiring hospitalization were reported (2 in HIIT, 1 in MICT); none were deemed related to the exercise interventions. | Not reported. |
|  |  |  |  |  |  |  |  |  |
| Bravo-Escobar R et al. (2017) Spain | RCT  NCT02796404 | Home-based CR (Mixed surveillance): 8-week program. 1 educational/training session per week at the hospital + 5 to 7 days of autonomous home-based walking sessions (70% HR reserve in month 1; 80% in month 2). Remotely monitored offline using a wearable ECG vest (NUUBO®). | Traditional CR (Control): 8-week standard center-based supervised CR at the hospital. | N=28. Patients with stable coronary artery disease (post-PCI or CABG) classified as having moderate cardiovascular risk. | 2 months | Both groups showed significant improvements in functional capacity (exercise time, METs) and quality of life (SF-36, Borg scale), with no significant differences between the modalities. | No major adverse cardiovascular events or exercise-related complications were recorded during the training sessions in either group. | Not reported. |
|  |  |  |  |  |  |  |  |  |
| Herring LY et al. (2021) UK | RCT  ISRCTN61195626 | PACES Intervention: A single 2.5-hour group-based structured education program focusing on physical activity, followed by automated text message support for 8 weeks. | Usual Care: Standard medical care, which may include referral to traditional center-based cardiac rehabilitation. | N=87 (mean age ~65 years, 82% male). Patients with diagnosed coronary heart disease (CHD). | 12 months | No significant between-group differences were observed in the primary outcome of physical activity or any secondary physiological and quality-of-life measures at 12 months. | No adverse events related to the intervention were reported. | Not reported. |
|  |  |  |  |  |  |  |  |  |
| Maddison R et al. (2014) New Zealand | Single-blind RCT  ACTRN12611000117910 | HEART Intervention: 24-week automated mobile phone program (118 tailored text messages) plus a secure website with role-model video vignettes, aimed at increasing exercise behavior to ≥ 30 mins most days . | Usual Care: Encouraged to participate in community-based CR, receive risk factor education, and join a local cardiac club. | N=171 (mean age 60.2, 81% male). Patients with stable ischemic heart disease (IHD) post-MI, angina, or revascularization within the previous 3-24 months. | 6 months | Although the intervention did not significantly improve exercise capacity (PVO2), it yielded significant gains in self-reported physical activity, walking, task self-efficacy, and general quality of life. | 31 serious adverse events (SAEs) were reported (15 cardiac-related); only 1 SAE (a cycling accident) was related to the study treatment. | Cardiac telerehabilitation demonstrates highly heterogeneous cost-effectiveness, spanning from dominant cost-saving strategies (e.g., an ICER of -€21,707/QALY) to economically non-viable programs burdened by unacceptably high ICERs (e.g., Aus $93,395.50/QALY) when initial setup costs are not offset by reduced healthcare utilization. |
|  |  |  |  |  |  |  |  |  |

**Supplementary Table S2. Head-to-head comparison of meta-analysis results under fixed/random-effects models**

| **Outcome or Subgroup** | | **Studies** | **Participants** | **Statistical Method** | **Effect Estimate** |
| --- | --- | --- | --- | --- | --- |
| SF-36 | Overall | 5 | 1264 | Mean Difference (IV, Fixed) I²=5% | MD=1.66, 95%CI [0.37, 2.96]; P=0.01 |
|  |  |  |  | Mean Difference (IV, Random) I²=5% | MD=1.39, 95%CI [-1.06, 3.84]; P=0.27 |
|  | Physical health-6m | 3 | 514 | Mean Difference (IV, Fixed) I²=4% | MD=0.43, 95%CI [-1.20, 2.07]; P=0.60 |
|  |  |  |  | Mean Difference (IV, Random) I²=4% | MD=0.43, 95%CI [-1.27, 2.13]; P=0.62 |
|  | Physical health-12m | 2 | 939 | Mean Difference (IV, Fixed) I²=0% | MD=1.48, 95%CI [0.71, 2.24]; P=0.0002 |
|  |  |  |  | Mean Difference (IV, Random) I²=0% | MD=1.48, 95%CI [0.71, 2.24]; P=0.0002 |
|  | Mental health-6m | 4 | 644 | Mean Difference (IV, Fixed) I²=0% | MD=1.73, 95%CI [0.22, 3.24]; P=0.02 |
|  |  |  |  | Mean Difference (IV, Random) I²=0% | MD=1.73, 95%CI [0.22, 3.24]; P=0.02 |
|  | Mental health-12m | 3 | 1069 | Mean Difference (IV, Fixed) I²=59% | MD=0.03, 95%CI [-0.92, 0.97]; P=0.95 |
|  |  |  |  | Mean Difference (IV, Random) I²=59% | MD=-0.10, 95%CI [-1.97, 1.76]; P=0.91 |
| HADS | Anxiety-6m | 5 | 1020 | Mean Difference (IV, Fixed) I²=0% | MD=-0.26, 95%CI [-0.66, 0.14]; P=0.21 |
|  |  |  |  | Mean Difference (IV, Random) I²=0% | MD=-0.26, 95%CI [-0.66, 0.14]; P=0.21 |
|  | Anxiety-12m | 2 | 210 | Mean Difference (IV, Fixed) I²=0% | MD=-0.24, 95%CI [-0.84, 0.37]; P=0.44 |
|  |  |  |  | Mean Difference (IV, Random) I²=0% | MD=-0.24, 95%CI [-0.84, 0.37]; P=0.44 |
|  | Depression-6m | 5 | 1024 | Mean Difference (IV, Fixed) I²=0% | MD=-0.17, 95%CI [-0.50, 0.17]; P=0.33 |
|  |  |  |  | Mean Difference (IV, Random) I²=0% | MD=-0.17, 95%CI [-0.50, 0.17]; P=0.33 |
|  | Depression-12m | 2 | 210 | Mean Difference (IV, Fixed) I²=0% | MD=-0.46, 95%CI [-1.18, 0.27]; P=0.22 |
|  |  |  |  | Mean Difference (IV, Random) I²=0% | MD=-0.46, 95%CI [-1.18, 0.27]; P=0.22 |
| MacNew | Physical score-6m | 2 | 433 | Mean Difference (IV, Fixed) I²=0% | MD=0.21, 95%CI [0.05, 0.38]; P=0.01 |
|  |  |  |  | Mean Difference (IV, Random) I²=0% | MD=0.21, 95%CI [0.05, 0.38]; P=0.01 |
|  | Emotional score-6m | 2 | 433 | Mean Difference (IV, Fixed) I²=0% | MD=0.16, 95%CI [-0.02, 0.33]; P=0.08 |
|  |  |  |  | Mean Difference (IV, Random) I²=0% | MD=0.16, 95%CI [-0.02, 0.33]; P=0.08 |
|  | Global score-6m | 3 | 563 | Mean Difference (IV, Fixed) I²=5% | MD=0.21, 95%CI [0.08, 0.33]; P=0.002 |
|  |  |  |  | Mean Difference (IV, Random) I²=5% | MD=0.21, 95%CI [0.07, 0.34]; P=0.002 |
|  | Physical score-12m | 1 | 90 | Mean Difference (IV, Fixed) I²=0% | MD=0.35, 95%CI [0.28, 0.42]; P＜0.00001 |
|  |  |  |  | Mean Difference (IV, Random) I²=0% | MD=0.35, 95%CI [0.28, 0.42]; P＜0.00001 |
|  | Emotional score-12m | 1 | 90 | Mean Difference (IV, Fixed) I²=0% | MD=0.35, 95%CI [-0.28, 0.42]; P＜0.00001 |
|  |  |  |  | Mean Difference (IV, Random) I²=0% | MD=0.35, 95%CI [-0.28, 0.42]; P＜0.00001 |
|  | Global score-12m | 2 | 220 | Mean Difference (IV, Fixed) I²=24% | MD=0.16, 95%CI [0.10, 0.22]; P＜0.00001 |
|  |  |  |  | Mean Difference (IV, Random) I²=24% | MD=0.17, 95%CI [0.07, 0.28]; P=0.0002 |
| MHLFQ | Overall | 3 | 244 | Mean Difference (IV, Fixed) I²=25% | MD=-5.99, 95%CI [-9.79, -2.18]; P=0.002 |
|  |  |  |  | Mean Difference (IV, Random) I²=25% | MD=-5.38, 95%CI [-10.29, -0.48]; P=0.03 |
| MACEs | Short-term | 4 | 442 | Risk Ratio (M-H, Fixed) I²=0% | RR=0.74, 95%CI [0.43, 1.27]; P=0.27 |
|  |  |  |  | Risk Ratio (M-H, Random) I²=0% | RR=0.73, 95%CI [0.43, 1.26]; P=0.26 |
|  | Medium-term | 4 | 487 | Risk Ratio (M-H, Fixed) I²=52% | RR=0.80, 95%CI [0.42, 1.53]; P=0.50 |
|  |  |  |  | Risk Ratio (M-H, Random) I²=52% | RR=0.81, 95%CI [0.27, 2.47]; P=0.71 |
|  | Long-term | 2 | 1556 | Risk Ratio (M-H, Fixed) I²=0% | RR=1.03, 95%CI [0.83, 1.28]; P=0.77 |
|  |  |  |  | Risk Ratio (M-H, Random) I²=0% | RR=1.03, 95%CI [0.83, 1.28]; P=0.77 |
| Cardiac rehospitalization | High risk | 4 | 1160 | Risk Ratio (M-H, Fixed) I²=77% | RR=0.78, 95%CI [0.66, 0.92]; P=0.004 |
|  |  |  |  | Risk Ratio (M-H, Random) I²=77% | RR=0.59, 95%CI [0.29, 1.21]; P=0.15 |
|  | Low risk | 10 | 2415 | Risk Ratio (M-H, Fixed) I²=52% | RR=0.88, 95%CI [0.75, 1.03]; P=0.11 |
|  |  |  |  | Risk Ratio (M-H, Random) I²=52% | RR=0.74, 95%CI [0.50, 1.09]; P=0.13 |
|  | Short-term | 4 | 410 | Risk Ratio (M-H, Fixed) I²=0% | RR=0.91, 95%CI [0.47, 1.76]; P=0.78 |
|  |  |  |  | Risk Ratio (M-H, Random) I²=0% | RR=0.94, 95%CI [0.48, 1.85]; P=0.86 |
|  | Medium-term | 4 | 531 | Risk Ratio (M-H, Fixed) I²=0% | RR=0.38, 95%CI [0.26, 0.56]; P＜0.00001 |
|  |  |  |  | Risk Ratio (M-H, Random) I²=0% | RR=0.38, 95%CI [0.26, 0.56]; P＜0.00001 |
|  | Long-term | 6 | 2634 | Risk Ratio (M-H, Fixed) I²=49% | RR=0.92, 95%CI [0.81, 1.04]; P=0.20 |
|  |  |  |  | Risk Ratio (M-H, Random) I²=49% | RR=0.91, 95%CI [0.71, 1.17]; P=0.47 |
|  | Software-Only Digital RCR | 5 | 614 | Risk Ratio (M-H, Fixed) I²=25% | RR=0.76, 95%CI [0.43, 1.34]; P=0.35 |
|  |  |  |  | Risk Ratio (M-H, Random) I²=25% | RR=0.77, 95%CI [0.35, 1.70]; P=0.51 |
|  | Hardware-Integrated Digital RCR | 8 | 1586 | Risk Ratio (M-H, Fixed) I²=70% | RR=0.73, 95%CI [0.63, 0.85]; P＜0.0001 |
|  |  |  |  | Risk Ratio (M-H, Random) I²=70% | RR=0.59, 95%CI [0.38, 0.92]; P=0.02 |
| All-cause mortality | | 5 | 1991 | Risk Ratio (M-H, Fixed) I²=0% | RR=1.02, 95%CI [0.73, 1.42]; P=0.91 |
|  |  |  |  | Risk Ratio (M-H, Random) I²=0% | RR=1.02, 95%CI [0.73, 1.43]; P=0.91 |
| All-cause adverse events | | 5 | 1018 | Risk Ratio (M-H, Fixed) I²=64% | RR=1.12, 95%CI [0.88, 1.43]; P=0.36 |
|  |  |  |  | Risk Ratio (M-H, Random) I²=64% | RR=1.19, 95%CI [0.62, 2.29]; P=0.60 |

**Supplementary Table S3. Subgroup of Studies Included in the Cardiac Rehospitalization Meta-Analysis**

| **Study (Author, Year)** | **Cardiovascular Risk Profile** | **Intervention Modality** | **Follow-up Duration** |
| --- | --- | --- | --- |
| Bernocchi P 2018 | High risk | Hardware-Integrated Digital | Medium |
| Chow CK 2022 | Low risk | Basic Telecommunication | Long term |
| Frederix I 2016 | Low risk | Hardware-Integrated Digital | Long term |
| Indraratna P 2022 | Low risk | Hardware-Integrated Digital | Medium |
| Kitsiou S 2025 | High risk | Hardware-Integrated Digital | Short term |
| Kraal JJ 2017 | Low risk | Hardware-Integrated Digital | Long term |
| Lao SSW 2023 | Low risk | Software-Only Digital | Short term |
| Li Z 2022 | Low risk | Software-Only Digital | Medium |
| Ögmundsdóttir Michelsen H 2022 | Low risk | Software-Only Digital | Long term |
| Pakrad F 2021 | Low risk | Software-Only Digital | Short term |
| Patterson K 2023 | Low risk | Hardware-Integrated Digital | Long term |
| Piotrowicz E 2020 | High risk | Hardware-Integrated Digital | Long term |
| Vetrovsky T 2024 | High risk | Hardware-Integrated Digital | Medium |
| Yudi MB 2021 | Low risk | Software-Only Digital | Short term |
